# Multifaceted Intensive Blood Pressure Control Model in Older and Younger Individuals With Hypertension: A Randomized Clinical Trial

**DOI:** 10.1001/jamacardio.2024.1449

**Published:** 2024-06-18

**Authors:** Xiaofan Guo, Nanxiang Ouyang, Guozhe Sun, Naijin Zhang, Zhao Li, Xingang Zhang, Guangxiao Li, Chang Wang, Lixia Qiao, Ying Zhou, Zihan Chen, Chuning Shi, Songyue Liu, Wei Miao, Danxi Geng, Pengyu Zhang, Yingxian Sun

**Affiliations:** 1 Department of Cardiology, First Hospital of China Medical University, Shenyang, China; 2 Department of Medical Record Management Center, First Hospital of China Medical University, Shenyang, China

## Abstract

**Importance:**

The sustainable effectiveness and safety of a nonphysician community health care practitioner–led intensive blood pressure intervention on cardiovascular disease have not, to the authors’ knowledge, been studied, especially in the older adult population.

**Objective:**

To evaluate such a multifaceted model with a more stringent blood pressure treatment goal (<130/80 mm Hg) among patients aged 60 years and older with hypertension.

**Design, Setting, and Participants:**

This was a 48-month follow-up study of the China Rural Hypertension Control Project (CRHCP), an open-cluster randomized clinical trial, conducted from 2018 to 2023. Participants 60 years and older and younger than 60 years with a diagnosis of hypertension from the CRHCP trial were included for analysis. Individuals were recruited from 326 villages in rural China.

**Interventions:**

The well-trained, nonphysician, community health care practitioner implemented a multifaceted intervention program (eg, initiation or titration of antihypertensive medications) to achieve a blood pressure level of less than 130/80 mm Hg, supervised by primary care physicians.

**Main Outcomes and Measures:**

Cardiovascular disease (a composite of myocardial infarction, stroke, heart failure requiring hospitalization, and cardiovascular disease death).

**Results:**

A total of 22 386 individuals 60 years and older with hypertension and 11 609 individuals younger than 60 years with hypertension were included in the analysis. The mean (SD) age of the participants was 63.0 (9.0) years and included 20 825 females (61.3%). Among the older individuals with hypertension, a total of 11 289 patients were randomly assigned to the intervention group and 11 097 to the usual-care group. During a median (IQR) of 4.0 (4.0-4.1) years, there was a significantly lower rate of total cardiovascular disease (1133 [2.7%] vs 1433 [3.5%] per year; hazard ratio [HR], 0.75; 95% CI, 0.69-0.81; *P* < .001) and all-cause mortality (1111 [2.5%] vs 1210 [2.8%] per year; HR, 0.90; 95% CI, 0.83-0.98; *P* = .01) in the intervention group than in the usual-care group. For patients younger than 60 years, the risk reductions were also significant for total cardiovascular disease (HR, 0.64; 95% CI, 0.56-0.75; *P* < .001), stroke (HR, 0.64; 95% CI, 0.55-0.76; *P* < .001), heart failure (HR, 0.39; 95% CI, 0.18-0.87; *P* = .02), and cardiovascular death (HR, 0.54; 95% CI, 0.37-0.77; *P* < .001), with all interaction *P* values for age groups greater than .05. In both age categories, the incidences of injurious falls, symptomatic hypotension, syncope, and the results for kidney outcomes did not differ significantly between groups.

**Conclusions and Relevance:**

In both the aging and younger general population with hypertension, the nonphysician health care practitioner–led, multifaceted, intensive blood pressure intervention model could effectively and safely reduce the risk of cardiovascular disease and all-cause death.

**Trial Registration:**

ClinicalTrials.gov Identifier: NCT03527719

## Key Points

**Question:**

Is it effective and safe to implement a nonphysician community health care practitioner–led, multifaceted, intensive blood pressure intervention in younger and older individuals with hypertension?

**Findings:**

This randomized clinical trial including 22 386 individuals 60 years and older and 11 609 individuals younger than 60 years demonstrated that such an intervention model sustainably and safely reduced incident cardiovascular disease by 25% and total mortality by 10% in older individuals with hypertension. The risk reductions were also profound in younger patients with hypertension.

**Meaning:**

This effective, feasible, and sustainable strategy should be integrated into hypertension control programs in low-resource settings in China and worldwide for both the older-age and younger population with hypertension.

## Teaser

This randomized clinical trial investigates the effectiveness and safety of a nonphysician-led, multifaceted, intensive blood pressure intervention in younger and older individuals with hypertension.

## Introduction

As the leading modifiable risk factor for cardiovascular disease (CVD) and all-cause death, hypertension is estimated to affect 33% of adults aged 30 to 79 years worldwide.^1,2,3^ Approximately 78% of adults with hypertension live in low- and middle-income countries (LMICs).^3^ Hypertension increases almost linearly with age, with a prevalence of around 60% over the age of 60 years and 75% over the age of 75 years.^4^ China has the largest number of older people worldwide.^5^ By 2040, the number of people 60 years and older is projected to be 402 million, making up nearly 30% of the national population.^6^ However, the treatment and control rate for hypertension in the older general population are low, especially in low-resource settings.^7,8^ An effective, safe, and sustainable strategy for hypertension management among the older population with higher CVD burden will have a substantial public health impact.

In recent years, several high-quality implementation studies showed promising results of nonphysician health care practitioner–led comprehensive intervention strategies on hypertension control.^9,10,11^ Nevertheless, they did not focus on older patients, and no CVD and death outcomes were evaluated. In the China Rural Hypertension Control Project (CRHCP) trial, we found that a nonphysician community health care practitioner–led intervention model was effective in reducing CVD outcomes.^12^ However, the sustainable effectiveness and safety of this multifaceted model among patients with hypertension who are 60 years and older has not been studied, to our knowledge.

The recent publication of the Strategy of Blood Pressure Intervention in the Elderly Hypertensive Patients (STEP) trial in patients aged 60 to 80 years has reignited the controversy of the optimal blood pressure (BP) target in older patients with hypertension.^13^ Together with the Systolic Blood Pressure Intervention Trial in Older Adults (SPRINT-SENIOR) trial, conducted in patients 75 years and older,^14^ it was found that more intensive BP control further lowered risk of CVD in the older population. These 2 randomized clinical trials need to be interpreted with caution because many patients with common medical conditions were strictly excluded. The effect of intensive BP control on CVD and death has not been established in the older general population with hypertension.

In the CRHCP trial, we have constructed a community-based, multifaceted intervention model led by nonphysician community health care practitioners (ie, village doctors, who work on the front line of basic primary care and public health service in rural China).^12,15^ Most village doctors have some medical training, such as 3 years of vocational or junior medical education. The large sample size of this trial provided a unique opportunity to conduct an independent study in a predefined subgroup of adults 60 years and older. In addition, results in participants younger than 60 years were available for comparison. We aimed to test the sustainable effectiveness and safety of such a multifaceted model with a more stringent BP treatment goal (<130/80 mm Hg) among the general population of patients with hypertension who are 60 years and older.

## Methods

### Study Design and Participants

The CRHCP is an open-label, blinded–end point, cluster randomized clinical trial conducted in 326 villages in rural China. The details of the design, eligibility, and baseline characteristics of CRHCP have been published previously (Supplement 1 and Supplement 2).^12,15^ Briefly, a total of 46 townships were randomly selected from 13 counties in 3 provinces (Liaoning, Shaanxi, and Hubei) in rural China. All eligible villages in each township were recruited. We conducted recruitment of all eligible patients with hypertension based on patient lists provided by village doctors in each village within the township. To better understand the demographic characteristics of the population, we collected information on ethnicity through questionnaires with self-reported answers from the patients. We primarily focused on the responses of Han, Manchu, and Mongolian ethnicities, as Han is the predominant ethnic group in China, and Manchu and Mongolian are more common in the population studied in this research. Written informed consent was provided by each participant at screening visits. The study was approved by the institutional review boards of the First Hospital of China Medical University and each participating site. This study followed the Consolidated Standards of Reporting Trials (CONSORT) reporting guidelines.

In the original design, the primary outcome was BP control at 18 months in phase 1 and CVD events over 36 months in phase 2. Before the end of phase 2 in October 2021, the CRHCP steering committee decided to extend the intervention and follow-up period to 48 months to test the sustainability and safety of the implementation strategy on dementia as well as CVDs. The randomization assignments for villages and individuals remained intact, and the intervention and control continued without interruption. In addition, using usual care as a control rather than a specified BP goal allowed for an ethical extension of the study. The revised CRHCP protocol was approved by the ethics committees at all participating institutes and by an independent data and safety monitoring board, which monitored the trial’s progress and reviewed safety and effectiveness data during the trial.

The trial included villages that had a regular nonphysician community health care practitioner and were at least 2 km away from one another. Eligible participants were aged at least 40 years, with at least 1 of the following criteria: mean untreated systolic BP of 140 mm Hg or higher and/or diastolic BP of 90 mm Hg or higher; mean treated systolic BP of 130 mm Hg or higher and/or diastolic BP of 80 mm Hg or higher for individuals without a history of clinical CVDs; or mean treated/untreated systolic BP of 130 mm Hg or higher and/or diastolic BP of 80 mm Hg or higher for individuals with a history of clinical CVD, diabetes, or chronic kidney disease from 6 measures on 2 different days. A full list of inclusion and exclusion criteria has been reported in the study protocol (Supplement 1).^16^

### Randomization and Masking

The randomization was undertaken at Tulane University Translational Science Institute in the US using SAS software (SAS Institute) stratified by provinces, counties, and townships. A biostatistician assigned all participating villages to intervention or usual care in a 1:1 ratio as per the random allocation sequence. The randomization assignments were concealed until recruitment and baseline data collection were completed. The end point adjudication committee members and the event adjudication coordinators were blinded to study assignment.

### Intervention

The nonphysician community health care practitioner–led multifaceted implementation strategy targeted health care systems, clinicians, patients, and communities (eTable 1 in Supplement 3). A simple stepped-care protocol for hypertension management was implemented based on clinical guidelines with a more stringent BP treatment goal (<130/80 mm Hg).^12,15^

The nonphysician community health care practitioner in the intervention group implemented the multifaceted implementation strategy. They received a series of training sessions covering details of daily hypertension management.^16^ After initial training and certification, retraining and quality improvement consultations were conducted periodically throughout the study.

The nonphysician community health care practitioner in the intervention group was responsible for routine treatment of patients with hypertension. For study-related tasks, the nonphysician community health care practitioner received a portion of their salaries from research grant funds and a performance-based incentive supplement. The primary care physicians at township hospitals guided and audited BP management and provided monthly feedback to the nonphysician community health care practitioners. Hypertension specialists at city or county hospitals provided routine training to nonphysician community health care practitioners and helped in treating patients with heart and kidney complications.

Discounted or free antihypertensive medications were provided to patients in the intervention villages monthly, the costs of which were covered by research grants. Patients in the intervention group also received free electronic home BP monitors and were trained on measuring and recording their BP 2 to 3 days a week to enhance medication adherence at the beginning of the study. Additionally, they received health coaching at individual visits or group sessions monthly in the first 6 months and quarterly thereafter. Social support groups among patients and family members were established in each intervention village.

Nonphysician community health care practitioners in the usual-care group did not receive hypertension management training or support, but they were trained on standard BP measurement. The patients in control villages received usual care from their nonphysician community health care practitioner or primary care physicians.

### Measurements

Demographic information was collected with the use of a standardized questionnaire at baseline. At each follow-up visit, BP was measured, and information was collected regarding lifestyle factors, antihypertensive medication use and adherence, health-related cost, and adverse events and trial outcomes. The 10-year risk for atherosclerotic CVD was calculated based on the American College of Cardiology and American Heart Association pooled cohort equations.^17^ An overnight fasting blood sample was collected in the morning from participants at baseline and 36-month follow-up visits to measure glucose, lipid, and electrolyte levels; liver and kidney function; and other routine blood biochemical indexes. The new Chronic Kidney Disease Epidemiology Collaboration creatinine equation was used to calculate estimated glomerular filtration rate.^18^ Three BP measurements were obtained at each visit according to a standard protocol using an automatic BP monitor (HBP-1100U [Omron]).^19^ The identification information and BP measurements of each participant were transmitted by mobile devices to the study data center in real time to avoid observer bias.

A standard questionnaire was used to identify CVD events and deaths in both groups. The process was supplemented by searching health insurance claims data from the China New Rural Cooperative Medical Scheme and death certificates from the local centers for disease control. After an event or death was identified, medical records were requested by study coordinators blinded to randomization assignments. A uniform cause of death questionnaire, adapted from validated verbal autopsy instruments in Chinese populations, was used for out of hospital deaths within 6 months.^20,21^

### Outcomes

The main outcome of the present study was total CVDs (a composite outcome of myocardial infarction, stroke, heart failure requiring hospitalization, and CVD death) during the 48-month follow-up. Other outcomes included myocardial infarction, stroke, heart failure requiring hospitalization, CVD death, all-cause death, and the composite of the CVD or all-cause death. Definitions and adjudication of study outcomes were reported in a previous article.^12^

Adverse events such as injurious falls, hypotension, and syncope were assessed systematically by trained research staff at every half-year visit using a standard questionnaire in both groups. Details of the safety outcomes were described previously.^12^

### Statistical Analysis

The number of clusters and participants was fixed for the CRHCP trial. Power to detect a 25% risk reduction associated with the intervention for the total CVD within the subgroup of participants 60 years and older was estimated assuming an enrollment of 163 clusters in each randomization group and 50 participants in each cluster. With a 4-year follow-up period, 2.4% per-year cardiovascular event rate (based on our previous trial data) in the control group, intracluster correlation coefficient within villages of 0.025 for cardiovascular disease, and a 2-sided significance level of .05, the statistical power was estimated to be 96%.

All analyses were based on the intention-to-treat approach. Cumulative event curves for the 2 trial groups were generated with the use of the Kaplan-Meier method, followed by a log-rank test. Marginal Cox proportional hazards models were used to estimate the hazard ratios (HRs) and 95% CIs of study outcomes associated with the intervention, in which the village was treated as a random effect and the stratification variables (province, county, and township) as fixed effects. A robust sandwich covariance matrix was used to account for village clusters. Follow-up time was censored on the date of loss to follow-up or last event ascertainment. We also adjusted for predefined baseline covariates in addition to the stratification variables. Exploratory secondary analyses were conducted to examine modification of the treatment effect by different subgroups. Two-sided tests at the .05 level were considered to be statistically significant. All analyses were performed using SAS, version 9.4 (SAS Institute), and R software (R Project for Statistical Computing).

## Results

A total of 22 386 individuals 60 years and older with hypertension and 11 609 individuals younger than 60 years with hypertension were included in the analysis. The mean (SD) age of the participants was 63.0 (9.0) years, 20 825 were female (61.3%), and 13 170 were male (38.7%). The population consisted of the following ethnicities: 31 022 Han (91.3%), 1444 Manchu (4.2%), 1435 Mongolian (4.2%), and 94 other ethnicities (0.3%). A total of 22 386 patients aged 60 years and older were randomly assigned to the intervention group (11 289 patients) or the usual-care group (11 097 patients) (eFigure 1 in Supplement 3), and 11 609 patients younger than 60 years were assigned to the intervention group (6118 patients) or usual-care group (5491 patients). The proportion of participants at a high risk of 10-year CVD was much higher among patients 60 years and older compared with those younger than 60 years, as expected (Table 1). Characteristics of participating villages were similar between the 2 groups (eTable 2 in Supplement 3).

**Table 1.** Baseline Characteristics of Participants 60 Years and Older and Younger Than 60 Years

Variable	No. (%)			
	≥60 y		<60 y	
	Intervention (n = 11 289)	Usual care (n = 11 097)	Intervention (n = 6118)	Usual care (n = 5491)
Age, mean (SD), y	68.2 (6.0)	68.3 (6.1)	52.8 (4.6)	52.9 (4.5)
Sex				
Female	6679 (59.2)	6611 (59.6)	3924 (64.1)	3611 (65.8)
Male	4610 (40.8)	4486 (40.4)	2194 (35.9)	1880 (34.2)
Education				
Primary school or lower	8495 (75.8)	8364 (76.3)	2899 (47.9)	2796 (51.3)
Junior high school	2231 (19.9)	2100 (19.2)	2574 (42.5)	2200 (40.4)
High school	470 (4.2)	470 (4.3)	475 (7.8)	385 (7.1)
College or higher	17 (0.2)	31 (0.3)	105 (1.7)	68 (1.2)
Cigarette smoking				
Never smoked	7807 (69.6)	7465 (68.1)	4340 (71.7)	3937 (72.3)
Former smokers	1098 (9.8)	1075 (9.8)	342 (5.7)	324 (5.9)
Current smokers	2316 (20.6)	2420 (22.1)	1371 (22.6)	1186 (21.8)
Weekly alcohol drinking	1650 (14.7)	1741 (15.9)	1143 (18.9)	946 (17.4)
Physical activity ≥5 times per wk^a^	4892 (43.7)	4953 (45.3)	3604 (59.6)	3280 (60.3)
Duration of hypertension, median (IQR), y	8 (5-13)	8 (5-12)	6 (4-9)	6 (4-9)
Use of antihypertensive medications	6735 (59.7)	5900 (53.2)	3839 (62.7)	3090 (56.3)
Antihypertensive medications, mean (SD), No. per patient	0.7 (0.7)	0.6 (0.7)	0.7 (0.7)	0.7 (0.7)
History of major cardiovascular disease^b^	2757 (24.4)	2557 (23.0)	955 (15.6)	820 (14.9)
History of stroke	2433 (21.6)	2262 (20.4)	848 (13.9)	712 (13.0)
History of diabetes	1110 (9.8)	991 (8.9)	475 (7.8)	435 (7.9)
History of chronic kidney disease	75 (0.7)	62 (0.6)	33 (0.5)	29 (0.5)
Body mass index, mean (SD)^c^	25.5 (3.8)	25.3 (3.8)	27.1 (3.8)	26.9 (3.7)
Systolic BP, mean (SD), mm Hg	158.3 (18.3)	156.9 (17.6)	154.4 (17.1)	152.5 (16.5)
Diastolic BP, mean (SD), mm Hg	85.7 (10.4)	85.1 (10.3)	92.5 (9.8)	91.7 (9.8)
Baseline BP level				
Systolic BP 130-139 mm Hg and/or diastolic BP 80-89 mm Hg	1385 (12.3)	1392 (12.5)	810 (13.2)	802 (14.6)
Systolic BP 140-159 mm Hg and/or diastolic BP 90-99 mm Hg	5268 (46.7)	5533 (49.9)	3018 (49.3)	2915 (53.1)
Systolic BP 160-179 mm Hg and/or diastolic BP 100-109 mm Hg	3202 (28.4)	2961 (26.7)	1656 (27.1)	1274 (23.2)
Systolic BP ≥180 mm Hg and/or diastolic BP ≥110 mm Hg	1434 (12.7)	1211 (10.9)	634 (10.4)	500 (9.1)
Criteria for enrollment into the study				
Untreated BP ≥140/90 mm Hg	3288 (29.1)	3792 (34.2)	1850 (30.2)	1961 (35.7)
Treated BP ≥130/80 mm Hg	4262 (37.8)	3830 (34.5)	2882 (47.1)	2295 (41.8)
Treated or untreated BP ≥130/80 mm Hg in those at high risk of cardiovascular disease^d^	3739 (33.1)	3475 (31.3)	1386 (22.7)	1235 (22.5)
Total cholesterol, mean (SD), mg/dL	195.9 (39.1)	194.8 (39.1)	193.5 (39.9)	193.6 (39.5)
LDL cholesterol, mean (SD), mg/dL	106.2 (31.9)	105.5 (31.7)	102.5 (32.3)	102.6 (31.5)
HDL cholesterol, mean (SD), mg/dL	56.5 (13.6)	56.3 (13.5)	54.7 (13.2)	54.3 (12.9)
Plasma glucose, mean (SD), mg/dL	111.6 (36.1)	111.3 (35.7)	110.8 (38.5)	109.9 (36.2)
Uric acid, mean (SD), mg/dL	5.1 (1.4)	5.1 (1.4)	5.1 (1.5)	5.1 (1.5)
eGFR, mean (SD), mL/min per 1.73 m^2 ^^e^	92.0 (12.1)	91.8 (11.9)	103.3 (10.9)	103.0 (10.8)
10-y Risk of atherosclerotic cardiovascular disease^f^				
Low risk (<20%)	4576 (57.3)	4636 (58.8)	4950 (97.0)	4499 (96.9)
High risk (≥20%)	3416 (42.7)	3254 (41.2)	154 (3.0)	146 (3.1)

Abbreviations: BP, blood pressure; eGFR, estimated glomerular filtration rate; HDL, high-density lipoprotein; LDL, low-density lipoprotein.

SI conversion factor: To convert HDL, LDL, and total cholesterol to millimoles per liter, multiply by 0.0259. To convert glucose millimoles per liter, multiply by 0.0555. To convert uric acid to millimoles per liter, multiply by 0.0595.

^a^ Moderate or heavy physical activity ≥30-minute each time.

^b^ Major cardiovascular disease includes myocardial infarction, stroke, and heart failure.

^c^ Calculated as weight in kilograms divided by height in meters squared.

^d^ Individuals with a history of clinical cardiovascular disease, diabetes, or chronic kidney disease.

^e^ eGFR was calculated on the basis of the 2021 Chronic Kidney Disease Epidemiology Collaboration creatinine equation.

^f^ Atherosclerotic cardiovascular disease risk was calculated with the American College of Cardiology/American Heart Association pooled population equation.

**Figure 1.**
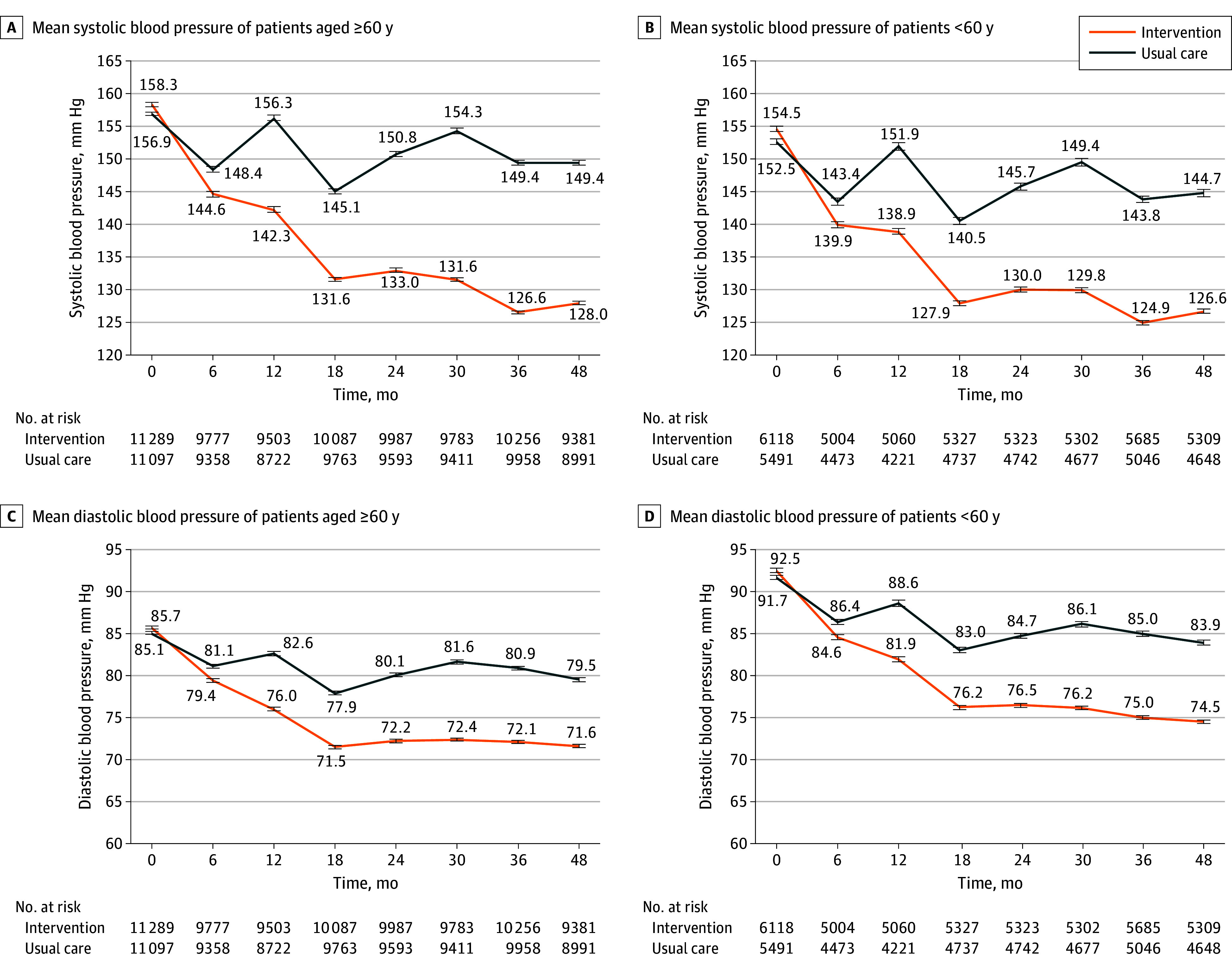
Systolic and Diastolic Blood Pressure in the Intervention and Usual-Care Groups Over the 48 Months of Follow-Up Among Patients 60 Years and Older and Younger Than 60 Years Mean systolic blood pressure among patients 60 years and older (A) and younger than 60 years (B). Mean diastolic blood pressure among patients 60 years and older (C) and younger than 60 years (D). Error bars represent 95% CIs.

Sustained between-group differences in both systolic and diastolic BP in older patients with hypertension were observed from 6 months on (Figure 1). Similar differences were observed in participants younger than 60 years and 80 years and older (Figure 1 and eFigure 2 in Supplement 3).

Overall, the incidence of total CVD was 2.2% per year in the intervention group and 3.0% in the usual-care group during a median (IQR) of 4.0 (4.0-4.1) years (HR, 0.72; 95% CI, 0.67-0.77; *P* < .001). The HR was 0.80 (95% CI, 0.66-0.97; *P* =  .02) for myocardial infarction, 0.71 (95% CI, 0.66-0.77; *P* <  .001) for stroke, 0.68 (95% CI, 0.54-0.85; *P* < .001) for heart failure, and 0.79 (95% CI, 0.75-0.84; *P* < .001) for CVD or death.

Among the older population with hypertension, total CVD occurred in 1133 participants (2.7% rate per year) in the intervention group, as compared with 1433 participants (3.5% rate per year) in the usual-care group (HR, 0.75; 95% CI, 0.69-0.81; *P* < .001) (Table 2). There were 1111 deaths from all causes (2.5% rate per year) in the intervention group and 1210 deaths from all causes (2.8% rate per year) in the usual-care group (HR, 0.90; 95% CI, 0.83-0.98; *P* = .01) (Table 2). The risk reductions were consistent for most of the outcomes except for myocardial infarction (Table 2 and eFigures 3-9 in Supplement 3). For example, participants in the intervention group had a lower risk of stroke (HR, 0.74; 95% CI, 0.68-0.81; *P* < .001), heart failure (HR, 0.73; 95% CI, 0.57-0.93; *P* = .01), and cardiovascular death (HR, 0.75; 95% CI, 0.65-0.85; *P* < .001). The results were similar after adjusting for multiple baseline risk factors (Table 2). For patients younger than 60 years, the incidence of total CVD was also lower in the intervention group than in the usual-care group (314 [1.3%] per year vs 424 [2.0%] per year) with a significant CVD risk reduction (HR, 0.64; 95% CI, 0.56-0.75; *P* < .001) (Table 2). The risk reductions were also profound for stroke (HR, 0.64; 95% CI, 0.55-0.76; *P* < .001), heart failure (HR, 0.39; 95% CI, 0.18-0.87; *P* = .02), and cardiovascular death (HR, 0.54; 95% CI, 0.37-0.77; *P* < .001) (Table 2 and eFigures 3-9 in Supplement 3).

**Table 2.** Effectiveness of a Nonphysician Community Health Care Practitioner–Led Intervention on Cardiovascular and Mortality Outcomes at 48 Months

Age, y	Intervention		Usual care		Hazard ratio (95% CI)^a^	*P* value^b^	Multiple-adjusted hazard ratio (95% CI)	*P* value^c^	*P* value for interaction
	Events, No.	Rate per year, %	Events, No.	Rate per year, %					
**Cardiovascular disease (myocardial infarction, stroke, heart failure, or cardiovascular disease death)**									
≥60	1133	2.7	1433	3.5	0.75 (0.69-0.81)	<.001	0.72 (0.67-0.78)	<.001	.11
<60	314	1.3	424	2.0	0.64 (0.56-0.75)	<.001	0.59 (0.51-0.68)	<.001	
**Myocardial infarction**									
≥60	136	0.3	156	0.4	0.85 (0.68-1.05)	.14	0.81 (0.65-1.02)	.07	.44
<60	38	0.2	48	0.2	0.69 (0.48-1.00)	.05	0.66 (0.45-0.96)	.03	
**Stroke**									
≥60	882	2.1	1121	2.7	0.74 (0.68-0.81)	<.001	0.72 (0.66-0.78)	<.001	.17
<60	269	1.1	363	1.7	0.64 (0.55-0.76)	<.001	0.59 (0.50-0.69)	<.001	
**Heart failure**									
≥60	98	0.2	127	0.3	0.73 (0.57-0.93)	.01	0.72 (0.56-0.91)	<.01	.15
<60	9	0.0	20	0.1	0.39 (0.18-0.87)	.02	0.36 (0.17-0.77)	<.01	
**Death from cardiovascular causes**									
≥60	315	0.7	406	1.0	0.75 (0.65-0.85)	<.001	0.73 (0.64-0.84)	<.001	.12
<60	48	0.2	77	0.4	0.54 (0.37-0.77)	<.001	0.49 (0.34-0.69)	<.001	
**Death from all causes**									
≥60	1111	2.5	1210	2.8	0.90 (0.83-0.98)	.01	0.90 (0.83-0.98)	.01	.21
<60	158	0.7	182	0.9	0.74 (0.61-0.90)	<.01	0.68 (0.56-0.84)	<.001	
**Cardiovascular disease or death**									
≥60 y	1899	4.5	2196	5.4	0.83 (0.78-0.88)	<.001	0.81 (0.76-0.86)	<.001	.04
<60 y	419	1.8	523	2.5	0.69 (0.61-0.78)	<.001	0.63 (0.56-0.71)	<.001	

^a^ 95% CIs were not adjusted for multiple comparisons and should not be used in place of hypothesis testing.

^b^ In the marginal Cox models, village was used as a random effect and the stratification variables (province, county, and township) as fixed effects.

^c^ Additionally adjusted for age, sex, cigarette smoking, use of antihypertensive medication, history of cardiovascular disease, and baseline systolic blood pressure, low-density lipoprotein cholesterol, and fasting plasma glucose.

There were no significant interactions between intervention and age groups stratified as 60 years and older and younger than 60 years for all studied outcomes except for CVD or death (Table 2). The risk reductions associated with intervention were further modeled as a function of age across the entire age spectrum. The magnitudes of risk reduction brought by intervention for CVD and death outcomes gradually diminished as age progressed (eFigure 10 in Supplement 3).

**Figure 2.**
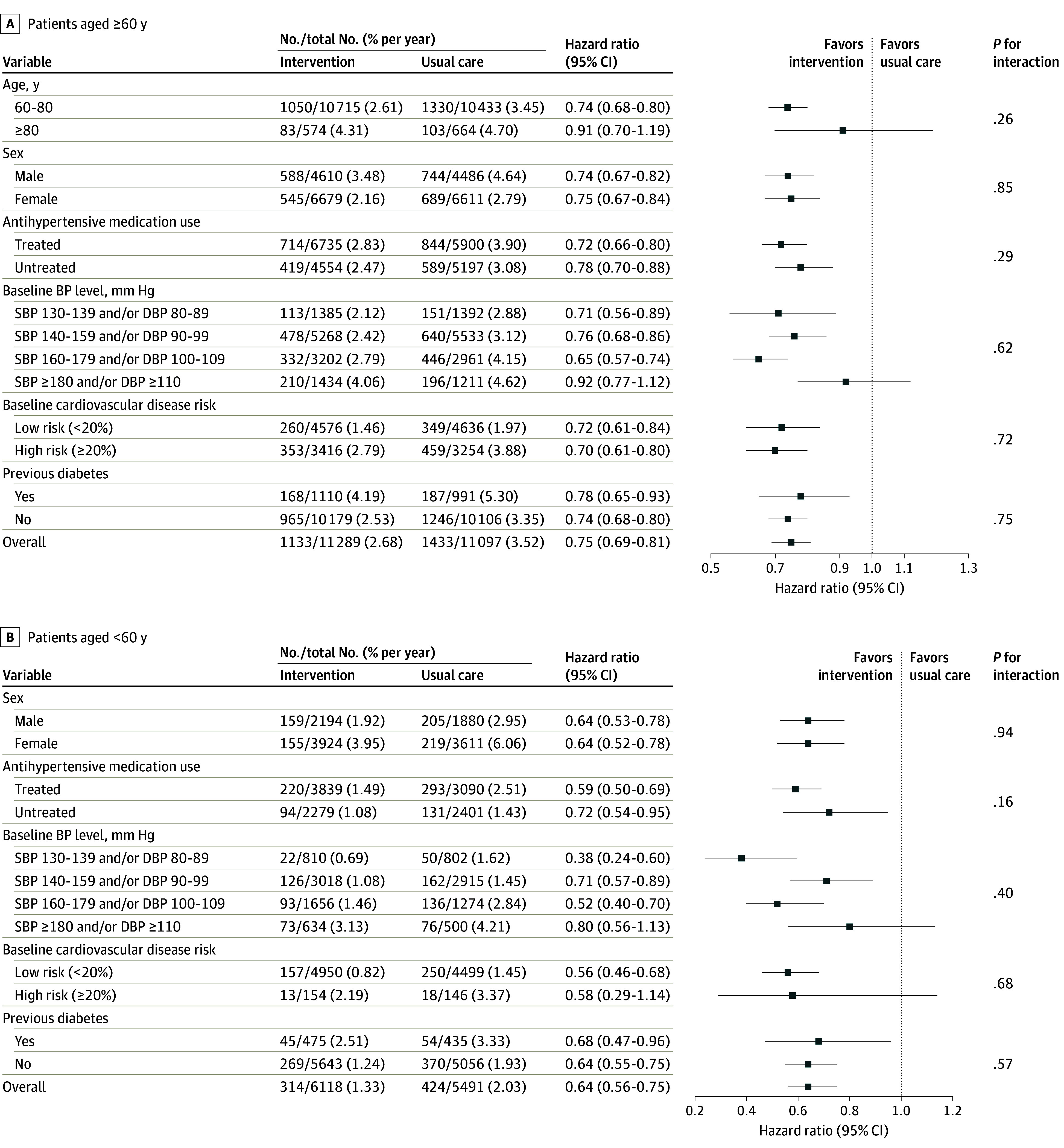
Forest Plot of Total Cardiovascular Disease According to Subgroups Among Patients 60 Years and Older and Younger Than 60 Years A, Patients 60 years and older. B, Patients younger than 60 years. DBP indicates diastolic blood pressure; SBP, systolic blood pressure.

Consistent results for total CVD were observed across different subgroups among participants 60 years and older and younger than 60 years (Figure 2). Analyses of the effects of intervention on other outcomes in subgroups of older and younger populations are shown in eFigures 11 to 16 in Supplement 3. The intraclass correlation coefficients of cardiovascular and mortality outcomes during 48-month follow-up were provided in eTable 3 in Supplement 3.

The incidences of injurious falls, syncope, and the results for kidney outcomes did not differ significantly between the 2 trial groups in both age segments (Table 3). For patients 60 years and older, although the rate of hypotension detected by BP measurements was higher in the intervention group, the rate of symptomatic hypotension resulting in a medical office visit was similar between groups. There was no significant difference in any hypotension between groups among patients younger than 60 years. Effectiveness and safety outcomes for patients 80 years and older are shown in eTables 4 and 5 in Supplement 3.

**Table 3.** Safety and Kidney Outcomes by Randomization Groups

Variable	No. (%)		Risk ratio (95% CI)	*P* value
	Intervention	Usual care		
**Patients aged ≥60 y**				
Conditions of interest				
Injurious falls^a^	69 (0.6)	58 (0.5)	1.17 (0.81-1.69)	.38
Hypotension^b^	225 (2.0)	90 (0.8)	2.45 (1.92-3.17)	<.001
Symptomatic hypotension^c^	88 (0.8)	70 (0.6)	1.24 (0.89-1.72)	.19
Syncope^d^	58 (0.5)	49 (0.4)	1.16 (0.78-1.74)	.44
Electrolytes^e^				
Serum sodium <130 mmol/L	6 (0.1)	5 (0.1)	1.16 (0.30-4.81)	.81
Serum sodium >150 mmol/L	45 (0.4)	152 (1.6)	0.29 (0.20-0.40)	<.001
Serum potassium <3.0 mmol/L	21 (0.2)	11 (0.1)	1.85 (0.85-4.25)	.09
Serum potassium >5.5 mmol/L	70 (0.7)	60 (0.6)	1.13 (0.79-1.62)	.49
Kidney outcomes^e^				
≥50% Reduction in estimated GFR in patients with chronic kidney disease at baseline, No./total No. (%)^f^	6/233 (2.6)	8/207 (3.9)	0.67 (0.19-2.19)	.46
≥30% Reduction in estimated GFR to <60 mL/min per 1.73 m^2^ in patients without chronic kidney disease at baseline, No./total No. (%)^f^	189/9841 (1.9)	152/9543 (1.6)	1.21 (0.97-1.50)	.09
**Patients aged <60 y**				
Conditions of interest				
Injurious falls^a^	24 (0.4)	24 (0.4)	0.90 (0.49-1.65)	.71
Hypotension^b^	79 (1.3)	58 (1.1)	1.22 (0.86-1.75)	.25
Symptomatic hypotension^c^	39 (0.6)	48 (0.9)	0.73 (0.47-1.14)	.14
Syncope^d^	14 (0.2)	15 (0.3)	0.84 (0.37-1.86)	.64
Electrolytes^e^				
Serum sodium <130 mmol/L	2 (0.0)	2 (0.0)	0.90 (0.07-12.38)	.92
Serum sodium >150 mmol/L	33 (0.6)	88 (1.8)	0.34 (0.22-0.51)	<.001
Serum potassium <3.0 mmol/L	15 (0.3)	7 (0.1)	1.92 (0.74-5.58)	.15
Serum potassium >5.5 mmol/L	18 (0.3)	32 (0.7)	0.50 (0.27-0.93)	.02
Kidney outcomes^e^				
≥50% Reduction in estimated GFR in patients with chronic kidney disease at baseline, No./total No. (%)^f^	2/35 (5.7)	2/34 (5.9)	0.97 (0.07-13.40)	.98
≥30% Reduction in estimated GFR to <60 mL/min per 1.73 m^2^ in patients without chronic kidney disease at baseline, No./total No. (%)^f^	39/5563 (0.7)	31/4919 (0.6)	1.11 (0.68-1.84)	.66

Abbreviation: GFR, glomerular filtration rate.

SI conversion factor: To convert serum potassium and sodium to milliequivalents per liter, divide by 1.

^a^ Self-reported injurious fall was defined as a fall that resulted in seeking medical care in a hospital, a primary care clinic, or a village doctor’s office.

^b^ Systolic blood pressure less than 90 mm Hg at a village doctor visit or a study data collection visit at months 6, 12, 18, 24, 30, 36, and 48.

^c^ Self-reported symptomatic hypotension was confirmed by systolic blood pressure less than 90 mm Hg at a village doctor visit.

^d^ Self-reported temporary loss of consciousness that resulted in seeking medical care in a hospital, a primary care clinic, or a village doctor’s office.

^e^ Results were based on 36-month follow-up.

^f^ Estimated GFR was calculated using the 2021 Chronic Kidney Disease Epidemiology Collaboration creatinine equations. Chronic kidney disease at baseline was defined as estimated GFR of less than 60 mL/min per 1.73 m^2^.

## Discussion

The present study extends and details the main CRHCP trial findings in community-based patients with hypertension who are 60 years and older and younger than 60 years in rural China, demonstrating that a nonphysician health care practitioner–led, multifaceted, intensive BP intervention model sustainably and safely reduced incident CVD by 25% and total mortality by 10% in the older general population. Meanwhile, we found that risk reductions of CVDs and mortality were also profound in the general population of patients with hypertension who are younger than 60 years. The benefit of intensive BP control was consistent among patients in both age ranges regardless of their baseline BP level, CVD risk category, or diabetes status.

By 2050, more than one-fifth of the world population will be aged 60 years and older, 80% of whom will be living in LMICs.^22^ The growing number combined with an increase in morbidity creates numerous challenges in the health care system in China.^23,24^ Our successfully implemented, multifaceted, intensive BP model should be scaled up throughout rural China as well as other low-resource settings worldwide. Although discounted or free antihypertensive medications may bring short-term economic stress, the benefits of CVD reduction demonstrated by our studies will outweigh the disadvantages. In addition, the medication costs of ¥588 ($85.30) per person over 3 years were very affordable.^12^ Our study findings call for health policy changes to make antihypertensive medications free and available to all patients in need.

Previous studies have shown that multicomponent strategies and task sharing with nonphysician health care workers are effective in reducing BP level.^9,10,25,26,27,28,29,30^ However, the majority of these studies were composed of small sample sizes and short implementation duration. With sound designs, the Control of Blood Pressure and Risk Attenuation—Bangladesh, Pakistan, and Sri Lanka (COBRA-BPS) trial and the health outcomes prevention and evaluation 4 (HOPE 4) project both adopted a more comprehensive strategy, but CVD and death outcomes were not available.^9,10^ Based on all the existing evidence, this randomized clinical trial developed an innovative multifaceted model, incorporating health care systems, health care practitioners, patients, and communities.^31,32^ The role of village doctors was remarkably amplified by provision with training, supervision, and performance-based financial incentives. We found that the intervention strategy gained sufficient benefit for patients with hypertension who are 60 years and older over a long period of 48 months, providing robust evidence for policymaking for the older adult population.

Although a few randomized clinical trials have investigated an intensive BP treatment goal on CVD in older patients with hypertension,^33,34,35,36,37^ the results are unclear in the general population. Both the SPRINT and STEP trials reported beneficial CVD outcomes among older patients with hypertension who are provided with intensive treatment.^13,14,38^ However, a list of conditions, such as diabetes and stroke, were strictly excluded in both trials. Making up for all these deficiencies, our trial for the first time, per our knowledge, provided strong evidence to support a BP treatment goal of less than 130/80 mm Hg in the older general population with hypertension in a community-based setting. Owing to a large sample size and diverse population included, we observed consistent reductions in stroke, heart failure, cardiovascular death, and all-cause death among the older population.

Safety is an important trade-off for intensive BP control, especially in older patients. The SPRINT-SENIOR study found comparable adverse effects between the 2 groups among patients aged 75 years or older,^14^ but the proportions of hypotension, syncope, and acute kidney injury or failure were significantly higher when using a cutoff of 60 years.^38^ Similar to the STEP trial,^13^ we found no significant differences between groups in symptomatic hypotension, injurious falls, and syncope among general older patients, except for hypotension reflected by BP readings. The kidney outcomes were also comparable between the 2 groups at 36 months.

Interestingly, we found that the risk reductions of cardiovascular and mortality outcomes were also significant in patients aged 40 to 60 years, without differences between the 2 groups in safety results, including hypotension. The Blood Pressure Lowering Treatment Trialists’ Collaboration (BPLTTC) also found worthwhile reductions in major cardiovascular events associated with BP reduction among participants younger than 65 years based on 51 randomized clinical trials.^39^ Our study provided a notably effective and safe model for hypertension management among younger population who has unique features of hypertension, such as higher diastolic BP as observed in the present study.

### Limitations

There are some limitations to the current study. Even though the trial had a large sample size and the statistical power was sufficient for the predefined subgroup of adults 60 years and older, randomization in CRHCP was not stratified by age categories. In addition, the trial did not collect information regarding frailty status. Frail, older patients tend to be associated with an increased vulnerability to many adverse health outcomes. However, the SPRINT trial showed that there was no evidence of heterogeneity for the cardiovascular benefit of intensive BP control by frailty or gait speed among the older adult population.^14,37^ Finally, we did not measure serum creatinine level at 48 months. However, there was no difference in kidney outcomes between the 2 groups at 3 years.

## Conclusions

In conclusion, results of this randomized clinical trial revealed that the nonphysician health care practitioner–led, multifaceted, BP intervention model with a treatment BP target of lower than 130/80 mm Hg could effectively and safely reduce risk of CVD and all-cause deaths in both the older and younger general population with hypertension. Specifically, these results have substantial implications for the future of hypertension management among older adults who represent a large proportion of the population and high absolute risk for CVD complications. This effective, feasible, and sustainable strategy should be integrated into hypertension control programs in low-resource settings in China and worldwide.

## Notes

Supplement 1.
Trial Protocol.

Supplement 2.
Statistical Analysis Plan.

Supplement 3. **eFigure 1.** Trial Profile for China Rural Hypertension Control Project (CRHCP) Participants Aged ≥ 60 Years and < 60 Years **eFigure 2.** Systolic and Diastolic Blood Pressure in the Intervention and Usual-Care Groups Over 48 Months Among Patients ≥ 80 Years **eFigure 3.** Kaplan-Meier Curves for Cardiovascular Disease Among Patients ≥ 60 Years and < 60 Years **eFigure 4.** Kaplan-Meier Curves for All-Cause Death Among Patients ≥ 60 Years and < 60 Years **eFigure 5.** Kaplan-Meier Curve for Myocardial Infarction Among Patients ≥ 60 Years and < 60 Years **eFigure 6.** Kaplan-Meier Curve for Stroke Among Patients ≥ 60 Years and < 60 Years **eFigure 7.** Kaplan-Meier Curve for Hospitalized Heart Failure Among Patients ≥ 60 Years and < 60 Years **eFigure 8.** Kaplan-Meier Curve for Death From Cardiovascular Causes Among Patients ≥ 60 Years and < 60 Years **eFigure 9.** Kaplan-Meier Curve for Cardiovascular Disease or Death Among Patients ≥ 60 Years and < 60 Years **eFigure 10.** Risk Reductions Associated With Intervention for Each Outcome as a Function of Age **eFigure 11.** Forest Plot of Death From All Causes According to Subgroups Among Patients ≥ 60 Years and < 60 Years **eFigure 12.** Forest Plot of Myocardial Infarction According to Subgroups Among Patients ≥ 60 Years and < 60 Years **eFigure 13.** Forest Plot of Stroke According to Subgroups Among Patients ≥ 60 Years and < 60 Years **eFigure 14.** Forest Plot of Hospitalized Heart Failure According to Subgroups Among Patients ≥ 60 Years and < 60 Years **eFigure 15.** Forest Plot of Death From Cardiovascular Causes According to Subgroups Among Patients ≥ 60 Years and < 60 Years **eFigure 16.** Forest Plot of Cardiovascular Disease or Death According to Subgroups Among Patients ≥ 60 Years and < 60 Years **eTable 1.** Implementation Strategies **eTable 2.** Characteristics of Clusters in the China Rural Hypertension Control Project **eTable 3.** Intraclass Correlation Coefficients (95% Confidence Intervals) of Cardiovascular and Mortality Outcomes During 48-Month Follow-Up **eTable 4.** Effectiveness of a Nonphysician Community Health Care Practitioner–Led Intervention on Cardiovascular and Mortality Outcomes Among Patients Aged ≥ 80 Years **eTable 5.** Safety and Kidney Outcomes by Randomization Groups Among Patients Aged ≥ 80 Years

Supplement 4. **Nonauthor Collaborators. **CRHCP Study Group.

Supplement 5.
Data Sharing Statement.
