# Supplementary material for: Multifaceted Intensive Blood Pressure Control Model in Older and Younger Individuals With Hypertension: A Randomized Clinical Trial
Source: JAMA Cardiol. 2024 Jun 18;9(9):781–90. doi: 10.1001/jamacardio.2024.1449 (PMC11195599; doi:10.1001/jamacardio.2024.1449)
Supplement: Supplement 2. — Statistical Analysis Plan. [file jamacardiol-e241449-s002.pdf]

China Rural Hypertension Control (CRHC) Project

Statistical Analysis Plan

November 2018

October 2020

October 2021

CRHC Project Study and Data Coordinating Center

The First Hospital of China Medical University, Shenyang, China

Tulane University Translational Science Institute, New Orleans, US

Section 1. Introduction

1. Background

Hypertension is the leading preventable risk factor for cardiovascular disease (CVD) and premature death in China.(1) The prevalence of hypertension is high and increasing while the control rate is low, especially in rural areas.(2-4) Uncontrolled hypertension plays an important role in the increased burden of CVD and stroke mortality in rural residents in China.(1) Traditionally, village doctors have played an important role in infectious disease control and delivering essential health services to rural residents in China.(5) With appropriate training and supervision, they could play an important role in hypertension control in rural China.(6)

2. Study Objectives

The overall objective of the CRHC Project is to develop an effective, feasible, and sustainable implementation strategy to achieve more intensive blood pressure (BP) control in rural residents in China. Moreover, this implementation trial will test the effectiveness of a lower BP target (<130/80 mmHg) on CVD outcomes. The specific aims for phases 1 and 2 of this project are:

Phase 1: to test whether a village doctor-led multifaceted intervention program will improve BP control (<130/80 mmHg) and reduce BP levels among hypertensive patients over an 18-month period compared to control.

Phase 2: to test whether a village doctor-led multifaceted intervention program will reduce CVD events, as well as mortality from CVD and all causes, among hypertensive patients over a 36-month period compared to control.

Phase 3: to test the effectiveness of a village doctor-led blood pressure intervention program compared to usual care on all-cause dementia and other cognitive impairment outcomes among patients with hypertension over a 48-month period.

Section 2. Study Methods

3. Study Design

The CRHC Project is a cluster randomized trial that will be conducted in 320 villages from three provinces in mainland China. One hundred and sixty villages will be randomly assigned to a village doctor-led multifaceted intervention and 160 villages to control, stratified by provinces, counties, and townships. A total of 32,000 individuals aged ≥40 years with uncontrolled hypertension will be recruited into the study. The village doctor-led multifaceted intervention is designed to overcome barriers at the healthcare system, provider, patient, and community levels.(7) Study participants are followed every 6 months for BP, CVD, and other study outcomes. The primary outcome is BP control (<130/80 mm Hg) at 18 months in phase 1, CVD events over 36 months in phase 2, and all-cause dementia over 48 months in phase 3.

4. Study Outcomes

Phase 1 will last for 18 months, phase 2 will last for 36 months, and phasse 3 will last for 48 minths. The primary and secondary study outcomes are as follows:

Phase 1 Study Outcomes

<u>Primary outcome:</u> Proportion of hypertension control (BP <130/80 mm Hg) at 18 months

<u>Secondary outcomes:</u>

- Changes in mean systolic and diastolic BP from baseline to 18 months
- Proportion of hypertension control (BP <140/90 mmHg)
- Proportion of patients adherent to antihypertensive medications at 18 months

Phase 2 Study Outcomes

Primary outcome: Composite cardiovascular disease (myocardial infarction, stroke, heart failure requiring hospitalization or treatment, and cardiovascular death)

Secondary outcome:

- Individual cardiovascular disease
  - Myocardial infarction
  - Stroke
  - Heart failure requiring hospitalization or treatment
  - Cardiovascular death
- All-cause mortality
- Changes in mean systolic and diastolic BP from baseline to 36 months
- Aortic dissection
- Incident malignant tumors

Phase 3 Study Outcomes

Primary outcome: All-cause dementia

Secondary outcome:

- Cognitive impairment no dementia
- Composite outcome of dementia or cognitive impairment no dementia
- Death from all causes
- Composite outcome of dementia or deaths
- Composite and individual cardiovascular disease (myocardial infarction, stroke, heart failure requiring hospitalization or treatment, and cardiovascular death)
- Changes in mean systolic and diastolic BP from baseline to 48 months
- Proportion of hypertension control (BP <130/80 mm Hg or <140/90 mmHg) at 48 months

5. Eligibility Criteria

Inclusion criteria for study villages:

- The village has a regular village doctor who is willing to participate in the hypertension control project
- The village does not plan to merge with other villages within 3 years
- The village is at least 2 kilometers away from other participating villages

- The village participates in the China New Rural Cooperative Medical Scheme

Inclusion criteria of study participants:

- Men or women aged ≥40 years
- Mean untreated systolic BP ≥140 mm Hg and/or diastolic BP ≥90 mm Hg or mean treated systolic BP ≥130 mm Hg and/or diastolic BP ≥80 mm Hg for individuals without a history of clinical CVD; or mean treated/untreated systolic BP ≥130 mm Hg and/or diastolic BP ≥80 mm Hg for individuals with a history of clinical coronary heart disease, heart failure, stroke, diabetes, or chronic kidney disease
- Have lived in a participating village for at least 6 months
- No intention to migrate within next 3 years
- Taking part in the New Rural Cooperative Medical Scheme
- Not pregnant or planning to become pregnant
- No malignant tumors and life expectancy ≥3 years
- Willing to participate and able to sign informed consent

6. Statistical Power and Sample Size

The sample size of the CRHC Project is calculated for the primary outcome of phase 2 due to the minimum required sample size being much larger in phase 2 than phase 1.

Phase 2 sample size:

The phase 2 sample size calculation is based on the following assumptions:

- 160 clusters in each of the intervention and control groups
- 2.0% per year CVD event rate in the control group
- 25% risk reduction associated with the intervention
- average follow-up duration of three years
- intra-cluster correlation coefficient (ICC) within villages of 0.025 for CVD events
- two-sided significance level of 0.05
- statistical power of 90%

The overall minimum sample size is 32,000 subjects (16,000 in each comparison group and 100 participants in each cluster) based on the Farrington & Manning Score test.(8) Loss to follow-up is not taking into consideration in sample size estimate due to availability of study villages and participants. However, we may elect to extend follow-up of participants for one more year if needed.

Phase 1 statistical power

Based on the sample size from phase 2, we have >99.9% statistical power to detect a 10% difference in the primary outcome between the two comparison groups based on the following assumptions:

- 20% of participants in the control group achieve BP <130/80 mmHg at 18 months
- 30% of participants in the intervention group achieve BP <130/80 mmHg at 18 months
- ICC of 0.05 for hypertension control.(9)

The statistical power is calculated using a Z test with a two-sided significance level of 0.05.(8)

Phase 3 statistical power

We calculated statistical power for phase 3 based on the following assumptions:

- 163 clusters in each group
- 104 participants per cluster
- 5.0% dementia event over 4 years
- 15% risk reduction
- average follow-up duration of 4 years
- lost-to-follow up rate of 1.6% per year
- intra-cluster correlation of 0.001, and
- 2-sided significance level of 0.05

Dementia event is based on previous studies from Chinese population (10,11) and effect size is based on a meta-analysis of results from the Syst-Eur (Systolic Hypertension in Europe) trial (12), the PROGRESS (Perindopril Protection Against Recurrent Stroke Study) trial (13), the HYVET (Hypertension in the Very Elderly) trial (14), and the SPRINT MIND (Systolic Blood Pressure Intervention Trial - Memory and Cognition in Decreased Hypertension) trial (15). The overall statistical power was determined to be 85.8%. The test statistic used is the two-sided Score test (Farrington & Manning) and implemented using PASS software (8).

Section 3. General Analysis Considerations

7. Timing of Analyses

The final analysis for phase 1 will be performed after all study participants have completed the 18-month visit 2; all study data have been transferred to the Study and Data Coordinating Center and passed quality control tests at the Tulane University Translational Science Institute; and this SAP document has been finalized and approved.

The final analysis for phase 2 will be performed after all study participants have completed the 36-month visit; all study data have been transferred to the Tulane University Translational Science Institute and documented as meeting the cleaning and quality requirements; and this SAP document has been finalized and approved.

The final analysis for phase 3 will be performed after all study participants have completed the 48-month visit; all study data have been transferred to the Tulane University Translational Science Institute and documented as meeting the cleaning and quality requirements; and this SAP document has been finalized and approved.

8. Analysis Populations

Intention-to-treat analyses will be conducted, in which study outcomes will be compared between participants according to their village randomization assignment, regardless of their actual adherence to the intervention.

9. Covariates and Subgroups

Co-variables will not be adjusted in the primary analysis. In a sensitivity analysis, important co-variables such as age, sex, education, duration of hypertension, and unbalanced CVD risk factors between the two comparison groups will be adjusted. Results from primary and sensitivity analyses will be compared. If they are consistent, the findings are less likely due to potential bias.

The pre-defined subgroup analyses will be conducted by the following covariables for Phase-1 and 2:

- age: <60 vs. ≥60 years
- sex: men vs. women
- education: <high school vs. ≥high school
- antihypertensive medication at baseline: use vs. no use
- cardiovascular risk: high risk (history of clinical myocardial infarction, stroke, or heart failure or atherosclerotic cardiovascular disease risk ≥20% based on ACC/AHA Pooled Population Equation) vs. low risk (<20%).(13)

The pre-defined subgroup analyses will be conducted by the following covariables for phase-3:

- age: median age of dementia participants
- sex: men vs. women
- education: median education level of dementia participants
- cigarette smoking: current smoking vs. not current smoking (never or former)
- body weight: median body mass index of dementia participants
- systolic blood pressure: median systolic blood pressure of dementia participants
- fasting plasma glucose: median fasting plasma glucose of dementia participants
- cardiovascular risk: median 10-year risk of atherosclerotic cardiovascular disease in dementia participants based on ACC/AHA Pooled Population Equation (16). Individuals with a history of clinical myocardial infarction, stroke, or heart failure will be categorized into the highest risk group.

10. Missing Data

In the primary analysis, no imputation will be used for missing data. In a secondary analysis, we will use multiple imputation for missing data in multivariable sensitivity analyses. The MCMC statement uses a Markov Chain Monte Carlo method to impute values for a data set with an arbitrary missing pattern, assuming a multivariate normal distribution for the data.(17,18) Point

estimates and confidence intervals will be compared from the complete case analysis and multiple imputation analysis.

11. Multiple Testing

The critical values will not be adjusted for in the primary and secondary study outcomes. There will be one primary study outcome in phase 1. Likewise, there will be one primary study outcome in phase 2 and phase 3.

The Bonferroni correction method will be used to adjust the critical value for interaction tests in the subgroup analyses. For example, a 2-sided p-value <0.003 (0.05/16) will be considered statistically significant for the ten tests in the subgroup analyses (19).

Section 4. Effectiveness and Other Analyses

12. Primary and Secondary Outcomes in Phase 1

In phase 1, we will test the difference in the proportions of patients with controlled BP between the two comparison groups using a generalized linear mixed-effects model:(20,21) $\log [P_{ij} /(1 - P_{ij})] = b0 + b1G_j + b2_j$, where P_{ij} denotes the probability of controlled BP for the ith participant, in the jth village at 18 months. The binary variable G_j indicates which randomization group is assigned to the jth village. The fixed effect b1 represents the intervention effect: the log-odds ratio of controlled BP associated with the intervention. $b2_j$ are the random effects at the village level with $b2_j \sim N(0, \upsilon^2)$, and the correlations of the outcome among the participants within each village are accounted for by assuming a compound symmetric correlation structure.

In addition, the difference in mean BP changes between the intervention and control groups will be tested using a linear mixed-effects model:(13,14) $Y_{ijl} = a0 + a1G_j + a2T_{ijl} + b1_j + b2_{ij} + e_{ijl}$, where Y_{ijl} is BP change from baseline for the ith participant nested in the jth village at the lth time. The fixed effect a1 represents the overall intervention effect across the study period, and a2 represents the time effect. We assume multilevel (nested) random effects at both village and patient levels, and they are represented by $b1_j$ with $b1_j \sim N(0, \upsilon^2)$ and $b2_{ij}$ with $b2_{ij} \sim N(0, \tau^2)$, respectively. The error, e_{ijl}, follows $N(0, \sigma^2)$. Rejection of the null hypothesis, a1=0, indicates a significant difference in BP change between the two comparison groups. Although an autoregressive correlation structure is the logical choice for these repeated measures, other correlation structures will be investigated as well.

13. Primary and Secondary Outcomes in Phase 2

In phase 2, the cumulative CVD event rates will be calculated using time-to-event methods according to randomization group and stratified by villages. The difference will be tested using a clustered log-rank test with the null hypothesis that cumulative incidences are the same between the two comparison groups.(22) Marginal structural Cox proportional hazards models will be used to assess the effectiveness of the village doctor-led intervention vs. control on CVD event rates stratified by village.(23,24) The hazard ratios and 95% confidence intervals associated with intervention effect will be presented.

Co-variables will not be adjusted in the primary analysis. In a sensitivity analysis, important co-variables such as age, sex, education, duration of hypertension, and unbalanced CVD risk factors between the two comparison groups will be adjusted. Results from the primary and sensitivity analyses will be compared. If they are consistent, the findings are less likely due to potential bias. In a sensitivity analysis, we will conduct multiple imputation for missing data, and

the findings from these analyses will be compared to those from the primary analysis without imputation.(25)

14. Primary and Secondary Outcomes in Phase 3

In phase 3, the proportions of all-cause dementia and secondary outcomes will be calculated according to the randomization group and stratified by township, county, and province. In this study, it is not possible to estimate the exact time of dementia onset because cognitive function tests are only conducted at the 48-month follow-up visit. Log-binomial and robust (or modified) Poisson regression models are most frequently applied to estimate the risk ratio for binary outcomes (26,27). Compared to the log-binomial model, robust Poisson regression provides unbiased estimates when the link function is mis-specified or when the probability distribution of the response variable is truncated at the right tail (28). In this trial, the risk ratio of village doctor-led intervention versus control on dementia event rates will be estimated using robust Poisson regression with a robust error variance, stratified by town, county, and province (28). Due to the clustered design, a compound symmetric working correlation structure will be utilized to account for the clustering effect within villages. SAS Proc GENMOD will be used for Poisson regression analysis.

Co-variables will not be adjusted in the primary analysis. In a sensitivity analysis, important co-variables such as age, sex, cigarette smoking, history of major cardiovascular disease, use of antihypertensive medication, systolic blood pressure, low-density lipoprotein cholesterol, and fasting plasma glucose at baseline will be adjusted.

15. Safety Analyses

Adverse events (AE) and serious adverse events (SAE) will be reported by comparison groups. Events will be reported as the number of and percentage of individuals experiencing an adverse event. Rates of adverse events will be compared using a χ^2 test.

16. Reference

1. He J, Gu D, Chen J, Wu X, Kelly TN, Huang JF, Chen JC, Chen CS, Bazzano LA, Reynolds K, Whelton PK, Klag MJ. Premature deaths attributable to blood pressure in China: a prospective cohort study. Lancet. 2009;374(9703):1765-72. Epub 2009/10/09. doi: 10.1016/s0140-6736(09)61199-5. PubMed PMID: 19811816.

2. Li Y, Yang L, Wang L, Zhang M, Huang Z, Deng Q, Zhou M, Chen Z, Wang L. Burden of hypertension in China: A nationally representative survey of 174,621 adults. Int J Cardiol. 2017;227:516-23. Epub 2016/11/20. doi: 10.1016/j.ijcard.2016.10.110. PubMed PMID: 27856040.

3. Lu J, Lu Y, Wang X, Li X, Linderman GC, Wu C, Cheng X, Mu L, Zhang H, Liu J, Su M, Zhao H, Spatz ES, Spertus JA, Masoudi FA, Krumholz HM, Jiang L. Prevalence, awareness, treatment, and control of hypertension in China: data from 1·7 million adults in a population-based screening study (China PEACE Million Persons Project). Lancet. 2017;390(10112):2549-58. Epub 2017/11/06. doi: 10.1016/s0140-6736(17)32478-9. PubMed PMID: 29102084.

4. Wang Z, Chen Z, Zhang L, Wang X, Hao G, Zhang Z, Shao L, Tian Y, Dong Y, Zheng C, Wang J, Zhu M, Weintraub WS, Gao R. Status of Hypertension in China: Results From the China Hypertension Survey, 2012-2015. Circulation. 2018;137(22):2344-56. Epub

2018/02/17. doi: 10.1161/circulationaha.117.032380. PubMed PMID: 29449338.

5. Li X, Lu J, Hu S, Cheng KK, De Maeseneer J, Meng Q, Mossialos E, Xu DR, Yip W, Zhang H, Krumholz HM, Jiang L, Hu S. The primary health-care system in China. Lancet. 2017;390(10112):2584-94. Epub 2017/12/13. doi: 10.1016/s0140-6736(17)33109-4. PubMed PMID: 29231837.

6. Long H, Huang W, Zheng P, Li J, Tao S, Tang S, Abdullah AS. Barriers and Facilitators of Engaging Community Health Workers in Non-Communicable Disease (NCD) Prevention and Control in China: A Systematic Review (2006⁻2016). Int J Environ Res Public Health. 2018;15(11). Epub 2018/10/31. doi: 10.3390/ijerph15112378. PubMed PMID: 30373205; PMCID: PMC6266440.

7. Sun Y, Li Z, Guo X, Zhou Y, Ouyang N, Xing L, Sun G, Mu J, Wang D, Zhao C, Wang J, Ye N, Zheng L, Chen S, Chang Y, Yang R, He J. Rationale and Design of a Cluster Randomized Trial of a Village Doctor-Led Intervention on Hypertension Control in China. Am J Hypertens. 2021;34(8):831-9. Epub 2021/02/20. doi: 10.1093/ajh/hpab038. PubMed PMID: 33605981.

8. Donner A, Klar N. Design and Analysis of Cluster Randomization Trials in Health Research. London: Arnold, 2000.

9. He J, Irazola V, Mills KT, Poggio R, Beratarrechea A, Dolan J, Chen CS, Gibbons L, Krousel-Wood M, Bazzano LA, Nejamis A, Gulayin P, Santero M, Augustovski F, Chen J, Rubinstein A. Effect of a Community Health Worker-Led Multicomponent Intervention on Blood Pressure Control in Low-Income Patients in Argentina: A Randomized Clinical Trial. Jama. 2017;318(11):1016-25. Epub 2017/10/05. doi: 10.1001/jama.2017.11358. PubMed PMID: 28975305; PMCID: PMC5761321.

10. Yuan J, Zhang Z, Wen H, Hong X, Hong Z, Qu Q, Tang M, Wu J, Xu Q, Li H, Cummings JL. Incidence of dementia and subtypes: A cohort study in four regions in China. Alzheimers Dement. 2016 Mar;12(3):262-71.

11. Jia L, Quan M, Fu Y, Zhao T, Li Y, Wei C, Tang Y, Qin Q, Wang F, Qiao Y, Shi S, Wang YJ, Du Y, Zhang J, Zhang J, Luo B, Qu Q, Zhou C, Gauthier S, Jia J; Group for the Project of Dementia Situation in China. Dementia in China: epidemiology, clinical management, and research advances. Lancet Neurol. 2020 Jan;19(1):81-92.

12. Forette F, Seux ML, Staessen JA, Thijs L, Birkenhäger WH, Babarskiene MR, Babeanu S, Bossini A, Gil-Extremera B, Girerd X, Laks T, Lilov E, Moisseyev V, Tuomilehto J, Vanhanen H, Webster J, Yodfat Y, Fagard R. Prevention of dementia in randomised double-blind placebo-controlled Systolic Hypertension in Europe (Syst-Eur) trial. Lancet. 1998 Oct 24;352(9137):1347-51. PMID: 9802273

13. Peters R, Beckett N, Forette F, Tuomilehto J, Clarke R, Ritchie C, Waldman A, Walton I, Poulter R, Ma S, Comsa M, Burch L, Fletcher A, Bulpitt C; HYVET investigators. Incident dementia and blood pressure lowering in the Hypertension in the Very Elderly Trial cognitive function assessment (HYVET-COG): a double-blind, placebo controlled trial. Lancet Neurol. 2008 Aug;7(8):683-9. PMID: 18614402

14. Tzourio C, Anderson C, Chapman N, Woodward M, Neal B, MacMahon S, Chalmers J. Effects of blood pressure lowering with perindopril and indapamide therapy on dementia

and cognitive decline in patients with cerebrovascular disease. Arch Intern Med 2003; 163(9): 1069-75.

15. Williamson JD, Pajewski NM, Auchus AP, Bryan RN, Chelune G, Cheung AK, Cleveland ML, Coker LH, Crowe MG, Cushman WC, Cutler JA, Davatzikos C, Desiderio L, Erus G, Fine LJ, Gaussoin SA, Harris D, Hsieh MK, Johnson KC, Kimmel PL, Tamura MK, Launer LJ, Lerner AJ, Lewis CE, Martindale-Adams J, Moy CS, Nasrallah IM, Nichols LO, Oparil S, Ogrocki PK, Rahman M, Rapp SR, Reboussin DM, Rocco MV, Sachs BC, Sink KM, Still CH, Supiano MA, Snyder JK, Wadley VG, Walker J, Weiner DE, Whelton PK, Wilson VM, Woolard N, Wright JT Jr, Wright CB. Effect of Intensive vs Standard Blood Pressure Control on Probable Dementia: A Randomized Clinical Trial. JAMA. 2019 Feb 12;321(6):553-561. PMID: 30688979

16. Goff DC, Jr., Lloyd-Jones DM, Bennett G, Coady S, D'Agostino RB, Sr., Gibbons R, Greenland P, Lackland DT, Levy D, O'Donnell CJ, Robinson JG, Schwartz JS, Shero ST, Smith SC, Jr., Sorlie P, Stone NJ, Wilson PWF. 2013 ACC/AHA guideline on the assessment of cardiovascular risk: a report of the American College of Cardiology/American Heart Association Task Force on Practice Guidelines. J Am Coll Cardiol. 2014;63(25 Pt B):2935-59. Epub 2013/11/19. doi: 10.1016/j.jacc.2013.11.005. PubMed PMID: 24239921; PMCID: PMC4700825.

17. He Y. Missing data analysis using multiple imputation: getting to the heart of the matter. Circulation Cardiovascular quality and outcomes. 2010;3(1):98-105. Epub 2010/02/04. doi: 10.1161/circoutcomes.109.875658. PubMed PMID: 20123676; PMCID: PMC2818781.

18. Sterne JA, White IR, Carlin JB, Spratt M, Royston P, Kenward MG, Wood AM, Carpenter JR. Multiple imputation for missing data in epidemiological and clinical research: potential and pitfalls. BMJ (Clinical research ed). 2009;338:b2393. Epub 2009/07/01. doi: 10.1136/bmj.b2393. PubMed PMID: 19564179; PMCID: PMC2714692.

19. Dunn OJ. Multiple Comparisons Among Means. J Am Stat Assoc. 1961;56(293):52–64.

20. Brown H, Prescott R. Applied Mixed Models in Medicine, 3rd Edition. New York: Wiley. 2015.

21. Diggle PJ, Liang KY, Zeger SL. Analysis of Longitudinal Data, 2nd Edition. Oxford: Oxford University Press, 2002.

22. Jung SH, Jeong JH. Rank tests for clustered survival data. Lifetime Data Anal. 2003;9(1):21-33. Epub 2003/02/27. doi: 10.1023/a:1021869803601. PubMed PMID: 12602772.

23. Cox DR. Regression models and life tables (with discussion). Journal of the Royal Statistical Society. 1972; Series B 34, 187-220. .

24. Lu SE, Wang MC. Marginal analysis for clustered failure time data. Lifetime Data Anal. 2005;11(1):61-79. Epub 2005/03/08. doi: 10.1007/s10985-004-5640-6. PubMed PMID: 15747590.

25. Bondarenko I, Raghunathan T. Graphical and numerical diagnostic tools to assess suitability of multiple imputations and imputation models. Stat Med. 2016;35(17):3007-20. Epub 2016/03/10. doi: 10.1002/sim.6926. PubMed PMID: 26952693.

26. Fitzmaurice GM, Lipsitz SR, Arriaga A, Sinha D, Greenberg C, Gawande AA. Almost efficient estimation of relative risk regression. Biostatistics. 2014;15(4):745-56.
27. Zou G. A modified Poisson regression approach to prospective studies with binary data. Am J Epidemiol. 2004;159(7):702-6.
28. Chen W, Qian L, Shi J, Franklin M. Comparing performance between log-binomial and robust Poisson regression models for estimating risk ratios under model misspecification. BMC Med Res Methodol. 2018;18, 63. https://doi.org/10.1186/s12874-018-0519-5.
