# Supplementary material for: Multifaceted Intensive Blood Pressure Control Model in Older and Younger Individuals With Hypertension: A Randomized Clinical Trial
Source: JAMA Cardiol. 2024 Jun 18;9(9):781–90. doi: 10.1001/jamacardio.2024.1449 (PMC11195599; doi:10.1001/jamacardio.2024.1449)
Supplement: Supplement 3. — eFigure 1. Trial Profile for China Rural Hypertension Control Project (CRHCP) Participants Aged ≥ 60 Years and < 60 Years eFigure 2. Systolic and Diastolic Blood Pressure in the Intervention and Usual-Care Groups Over 48 Months Among Patients ≥ 80 Years eFigure 3. Kaplan-Meier Curves for Cardiovascular Disease Among Patients ≥ 60 Years and < 60 Years eFigure 4. Kaplan-Meier Curves for All-Cause Death Among Patients ≥ 60 Years and < 60 Years eFigure 5. Kaplan-Meier Curve for Myocardial Infarction Among Patients ≥ 60 Years and < 60 Years eFigure 6. Kaplan-Meier Curve for Stroke Among Patients ≥ 60 Years and < 60 Years eFigure 7. Kaplan-Meier Curve for Hospitalized Heart Failure Among Patients ≥ 60 Years and < 60 Years eFigure 8. Kaplan-Meier Curve for Death From Cardiovascular Causes Among Patients ≥ 60 Years and < 60 Years eFigure 9. Kaplan-Meier Curve for Cardiovascular Disease or Death Among Patients ≥ 60 Years and < 60 Years eFigure 10. Risk Reductions Associated With Intervention for Each Outcome as a Function of Age eFigure 11. Forest Plot of Death From All Causes According to Subgroups Among Patients ≥ 60 Years and < 60 Years eFigure 12. Forest Plot of Myocardial Infarction According to Subgroups Among Patients ≥ 60 Years and < 60 Years eFigure 13. Forest Plot of Stroke According to Subgroups Among Patients ≥ 60 Years and < 60 Years eFigure 14. Forest Plot of Hospitalized Heart Failure According to Subgroups Among Patients ≥ 60 Years and < 60 Years eFigure 15. Forest Plot of Death From Cardiovascular Causes According to Subgroups Among Patients ≥ 60 Years and < 60 Years eFigure 16. Forest Plot of Cardiovascular Disease or Death According to Subgroups Among Patients ≥ 60 Years and < 60 Years eTable 1. Implementation Strategies eTable 2. Characteristics of Clusters in the China Rural Hypertension Control Project eTable 3. Intraclass Correlation Coefficients (95% Confidence Intervals) of Cardiovascular and Mortality Outcomes During 48-Month Follow-Up eTable 4. Eff [file jamacardiol-e241449-s003.pdf]

Supplementary Online Content

Guo X, Ouyang N, Sun G, et al; CRHCP Study Group. Multifaceted intensive blood pressure control model in older and younger individuals with hypertension: a randomized clinical trial. *JAMA Cardiol*. Published online June 18, 2024. doi:10.1001/jamacardio.2024.1449

eFigure 1. Trial Profile for China Rural Hypertension Control Project (CRHCP) Participants Aged ≥ 60 Years and < 60 Years

eFigure 2. Systolic and Diastolic Blood Pressure in the Intervention and Usual-Care Groups Over 48 Months Among Patients ≥ 80 Years

eFigure 3. Kaplan-Meier Curves for Cardiovascular Disease Among Patients ≥ 60 Years and < 60 Years

eFigure 4. Kaplan-Meier Curves for All-Cause Death Among Patients ≥ 60 Years and < 60 Years

eFigure 5. Kaplan-Meier Curve for Myocardial Infarction Among Patients ≥ 60 Years and < 60 Years

eFigure 6. Kaplan-Meier Curve for Stroke Among Patients ≥ 60 Years and < 60 Years

eFigure 7. Kaplan-Meier Curve for Hospitalized Heart Failure Among Patients ≥ 60 Years and < 60 Years

eFigure 8. Kaplan-Meier Curve for Death From Cardiovascular Causes Among Patients ≥ 60 Years and < 60 Years

eFigure 9. Kaplan-Meier Curve for Cardiovascular Disease or Death Among Patients ≥ 60 Years and < 60 Years

eFigure 10. Risk Reductions Associated With Intervention for Each Outcome as a Function of Age

eFigure 11. Forest Plot of Death From All Causes According to Subgroups Among Patients ≥ 60 Years and < 60 Years

eFigure 12. Forest Plot of Myocardial Infarction According to Subgroups Among Patients ≥ 60 Years and < 60 Years

eFigure 13. Forest Plot of Stroke According to Subgroups Among Patients ≥ 60 Years and < 60 Years

© 2024 Guo X et al. *JAMA Cardiology*.

eFigure 14. Forest Plot of Hospitalized Heart Failure According to Subgroups Among Patients ≥ 60 Years and < 60 Years

eFigure 15. Forest Plot of Death From Cardiovascular Causes According to Subgroups Among Patients ≥ 60 Years and < 60 Years

eFigure 16. Forest Plot of Cardiovascular Disease or Death According to Subgroups Among Patients ≥ 60 Years and < 60 Years

eTable 1. Implementation Strategies

eTable 2. Characteristics of Clusters in the China Rural Hypertension Control Project

eTable 3. Intraclass Correlation Coefficients (95% Confidence Intervals) of Cardiovascular and Mortality Outcomes During 48-Month Follow-Up

eTable 4. Effectiveness of a Nonphysician Community Health Care Practitioner–Led Intervention on Cardiovascular and Mortality Outcomes Among Patients Aged ≥ 80 Years

eTable 5. Safety and Kidney Outcomes by Randomization Groups Among Patients Aged ≥ 80 Years

This supplementary material has been provided by the authors to give readers additional information about their work.

© 2024 Guo X et al. *JAMA Cardiology*.

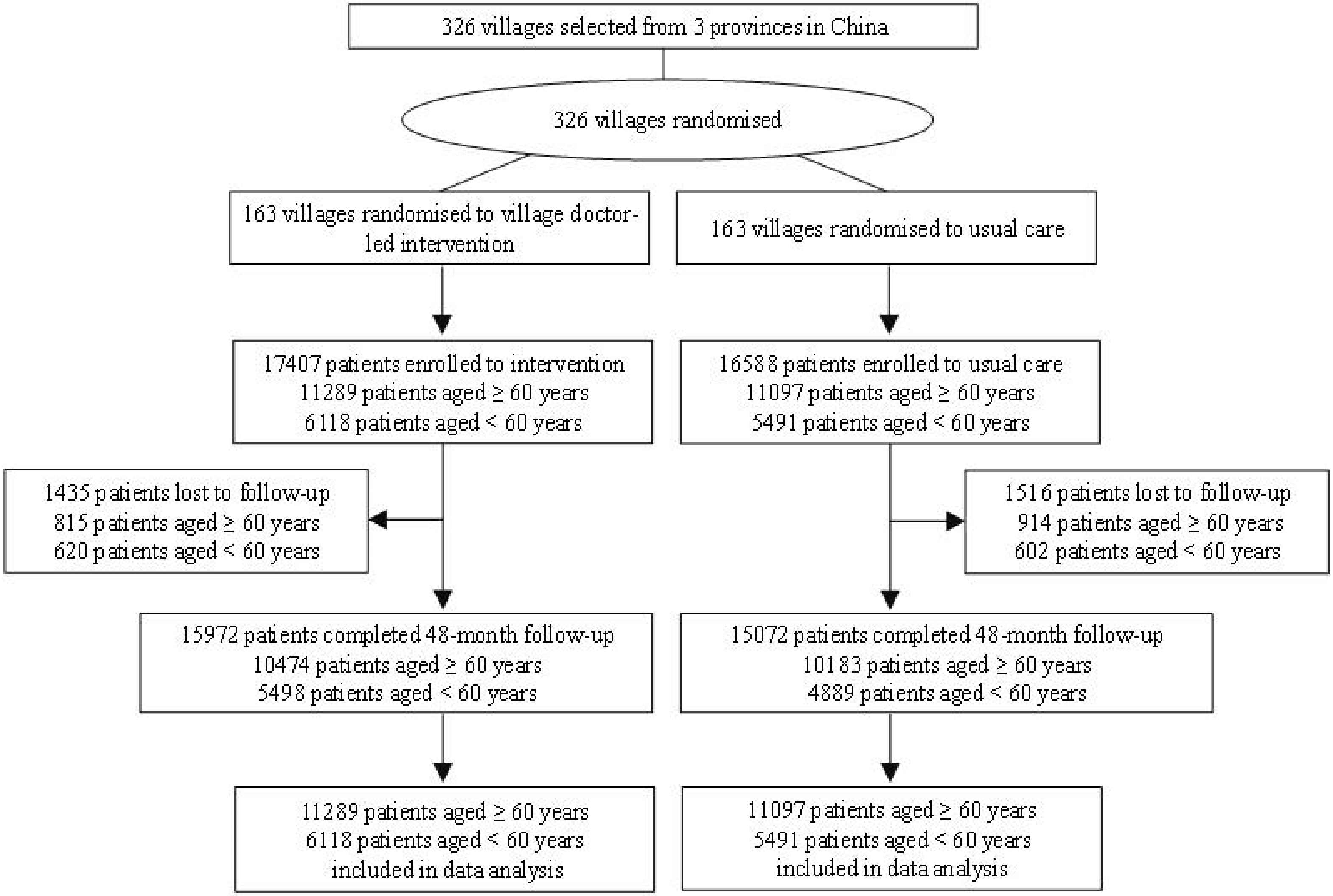

eFigure 1: Trial profile for China Rural Hypertension Control Project (CRHCP) participants aged ≥ 60 years and < 60 years.

© 2024 Guo X et al. *JAMA Cardiology*.

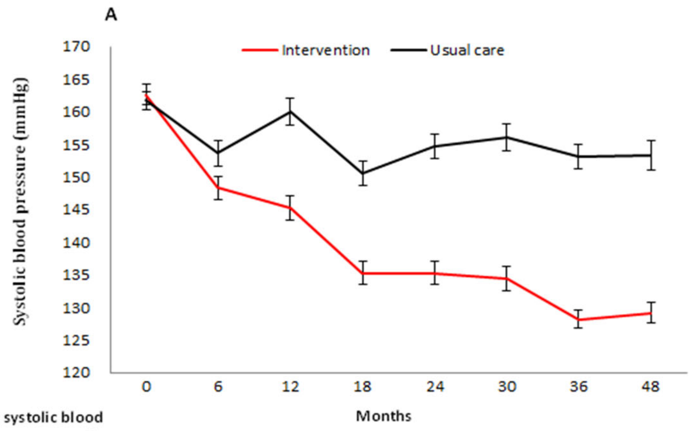

Mean systolic blood pressure, mm Hg	0	6	12	18	24	30	36	48
Intervention	162.6	148.3	145.2	135.3	135.3	134.4	128.2	129.2
Usual care	161.7	153.6	160	150.6	154.7	156.1	153.1	153.3

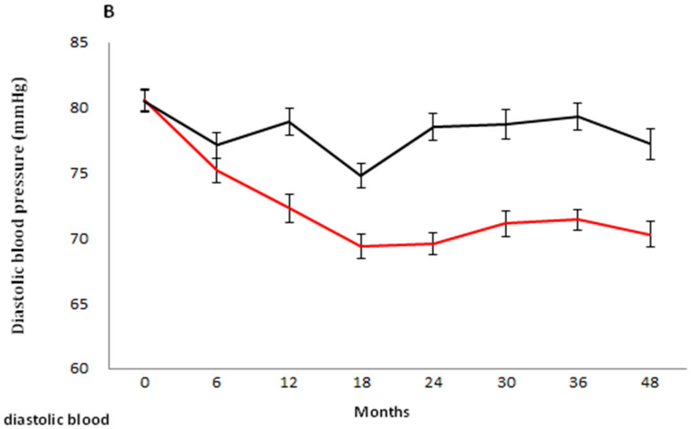

Mean diastolic blood pressure, mm Hg	0	6	12	18	24	30	36	48
Intervention	80.6	75.2	72.3	69.4	69.6	71.1	71.4	70.3
Usual care	80.5	77.1	78.9	74.8	78.5	78.7	79.3	77.2

Number of participants with data	0	6	12	18	24	30	36	48
Intervention	574	463	412	453	445	437	454	341
Usual care	664	527	438	507	493	472	495	383

eFigure 2: Systolic and diastolic blood pressure in the intervention and usual care groups over 48 months among patients ≥ 80 years.

© 2024 Guo X et al. *JAMA Cardiology*.

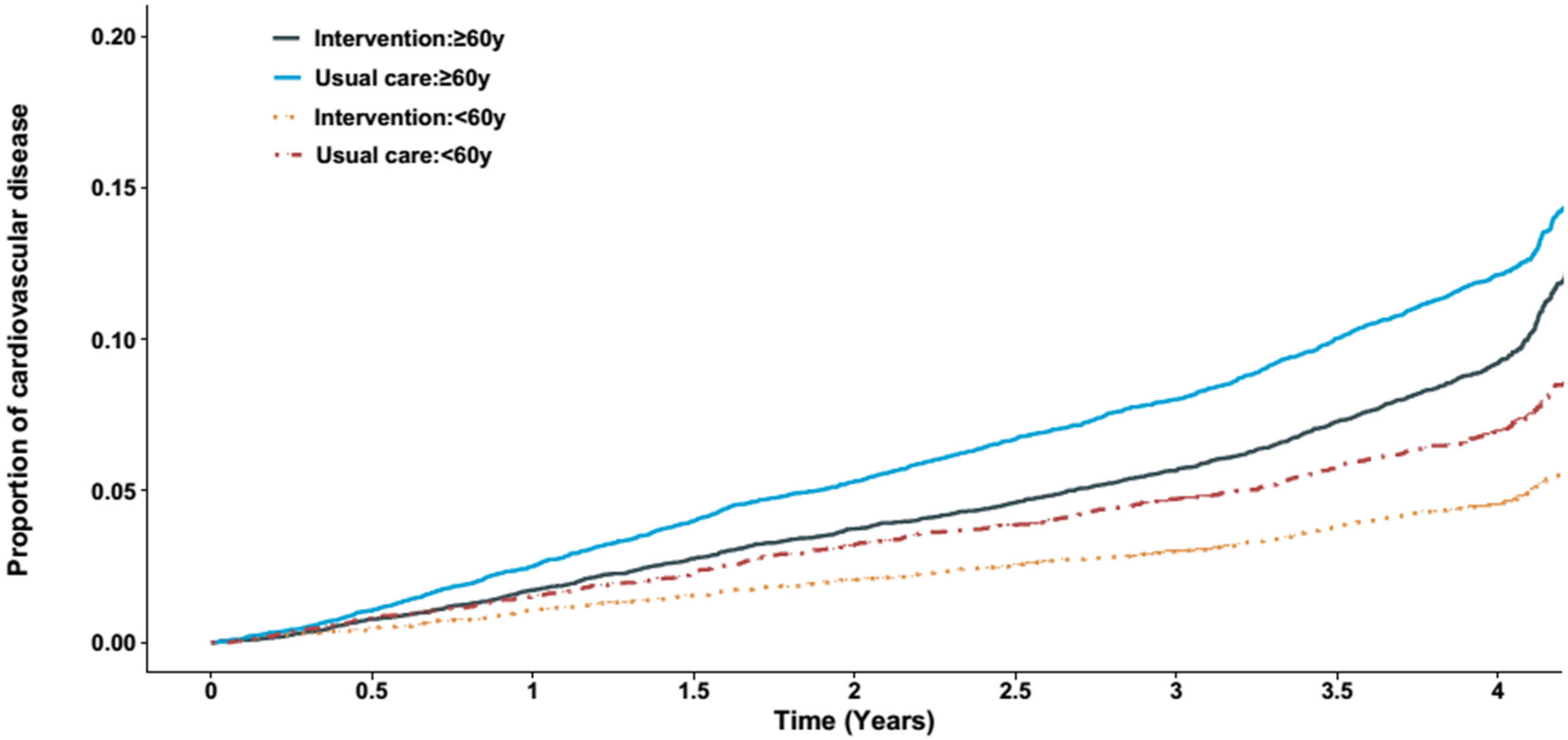

Number at risk

Intervention:≥60y	11258	11141	10916	10697	10511	10298	9741	9015	6423
Intervention:<60y	6092	6055	5989	5933	5875	5803	5521	5158	3843
Usual care:≥60y	11083	10925	10655	10397	10184	9897	9266	8459	6189
Usual care:<60y	5469	5421	5352	5295	5226	5148	4871	4466	3363

eFigure 3: Kaplan-Meier curves for cardiovascular disease among patients ≥ 60 years and < 60 years.

© 2024 Guo X et al. *JAMA Cardiology*.

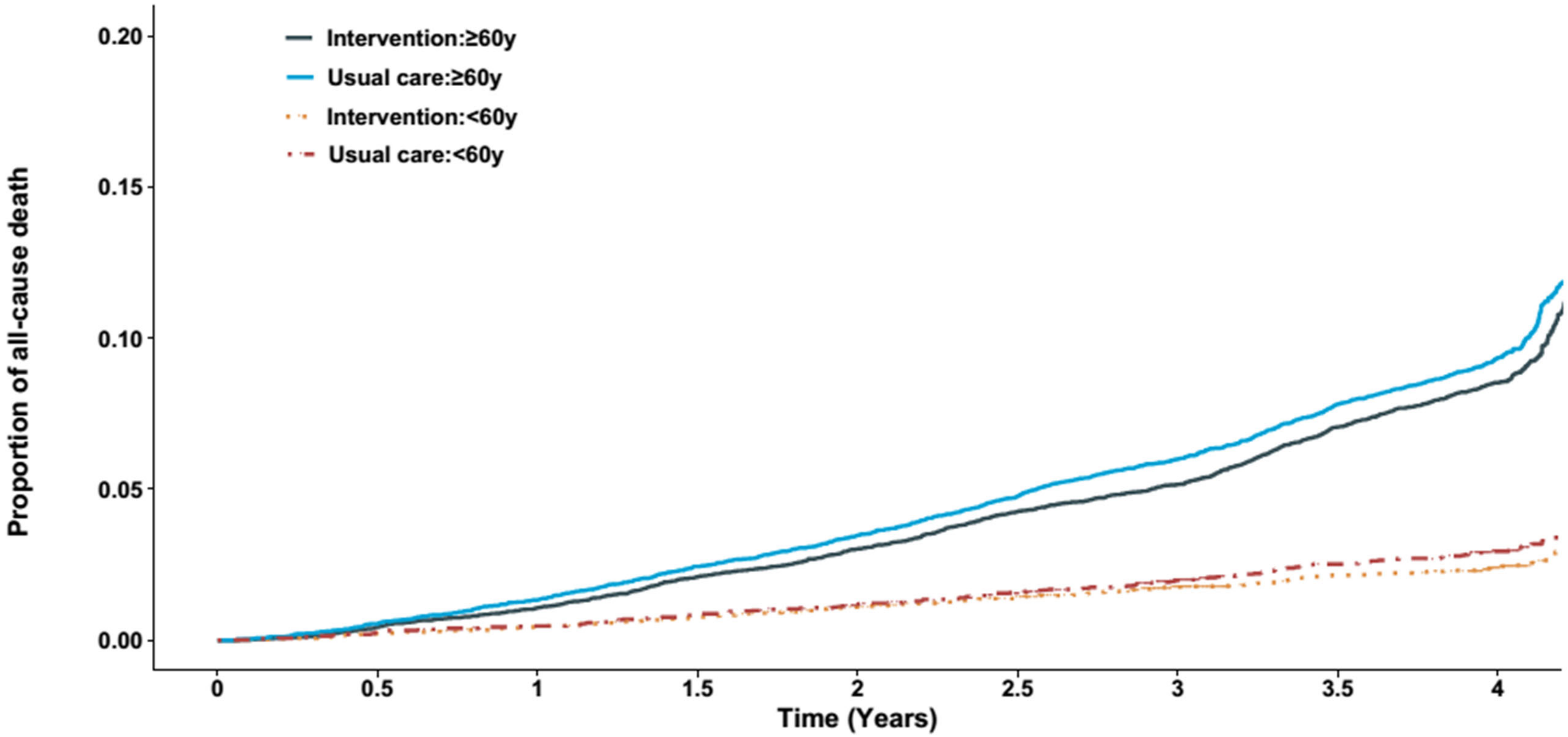

Number at risk

Intervention:≥60y	11258	11207	11120	10992	10878	10702	10273	9620	6899
Intervention:<60y	6092	6078	6054	6024	5987	5935	5665	5329	3975
Usual care:≥60y	11083	11022	10918	10787	10657	10468	9944	9242	6861
Usual care:<60y	5469	5455	5434	5409	5375	5319	5073	4684	3555

eFigure 4: Kaplan-Meier curves for all-cause death among patients ≥ 60 years and < 60 years.

© 2024 Guo X et al. *JAMA Cardiology*.

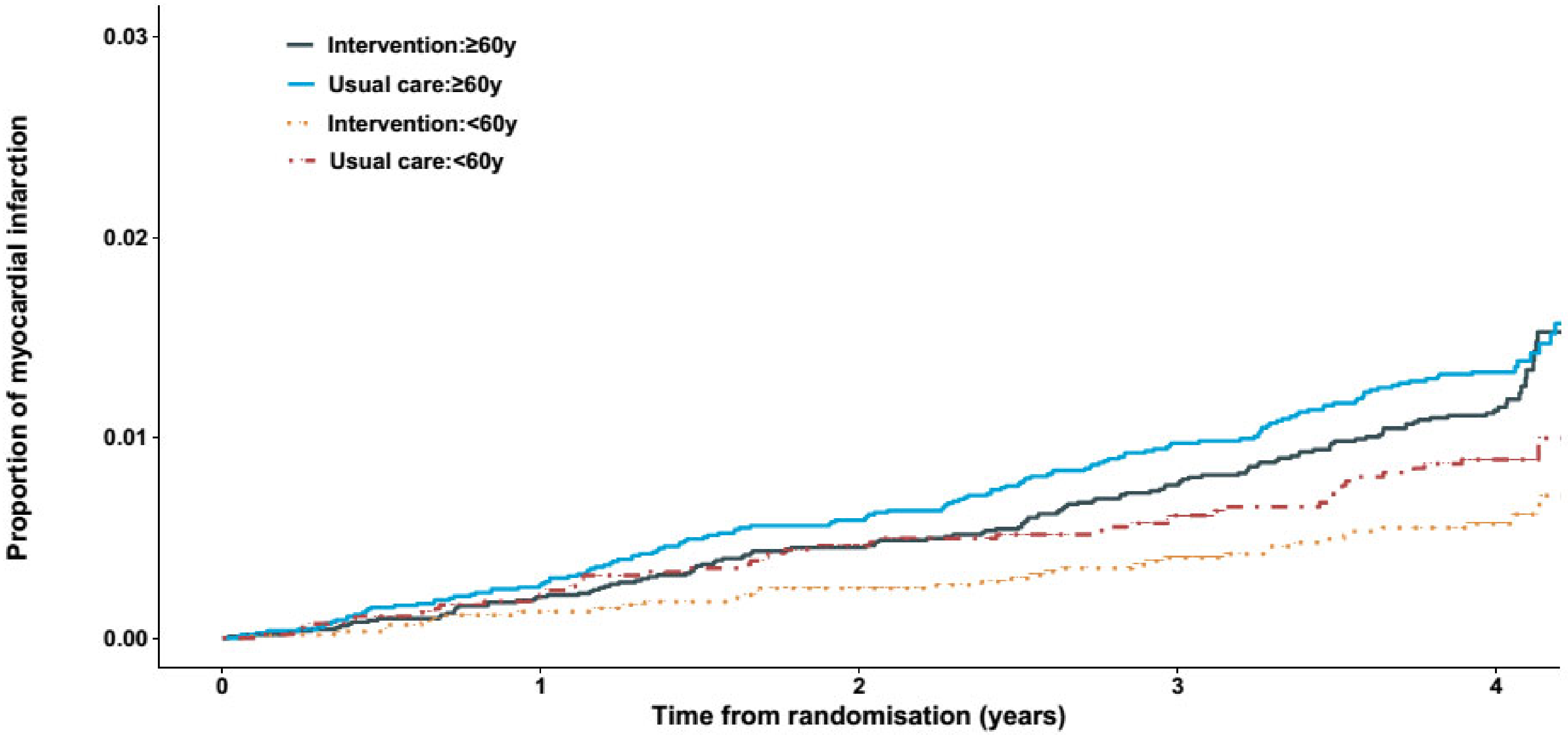

Number at risk

	0	1	2	3	4
Intervention:≥60y	11258	11047	10783	10056	6727
Intervention:<60y	6092	6036	5966	5631	3942
Usual care:≥60y	11083	10833	10566	9739	6655
Usual care:<60y	5469	5412	5349	5028	3512

eFigure 5: Kaplan-Meier curve for myocardial infarction among patients ≥ 60 years and < 60 years.

© 2024 Guo X et al. *JAMA Cardiology*.

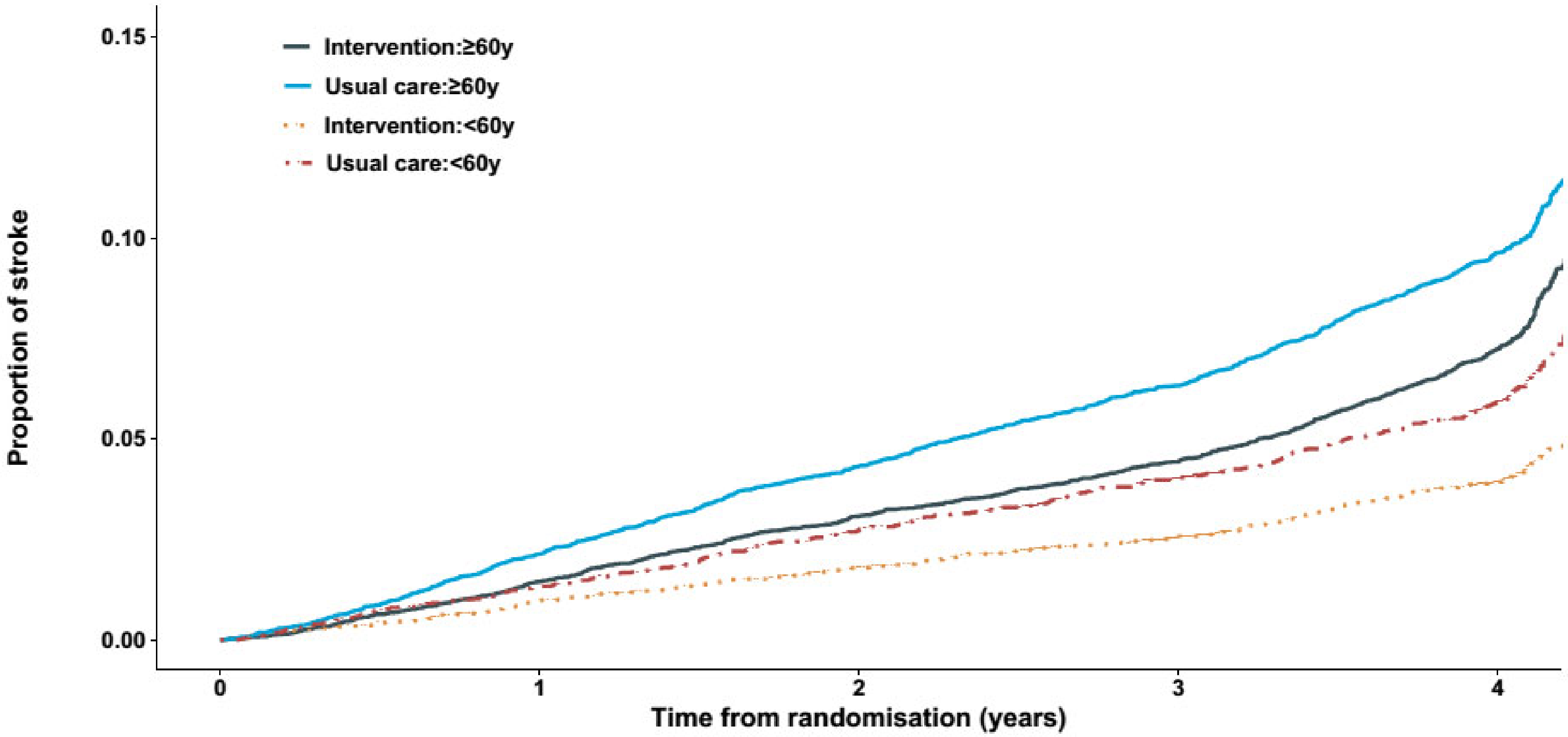

Number at risk

	0	1	2	3	4
Intervention:≥60y	11258	10935	10549	9798	6478
Intervention:<60y	6092	5995	5887	5541	3861
Usual care:≥60y	11083	10668	10242	9350	6269
Usual care:<60y	5469	5361	5246	4898	3394

eFigure 6: Kaplan-Meier curve for stroke among patients ≥ 60 years and < 60 years.

© 2024 Guo X et al. *JAMA Cardiology*.

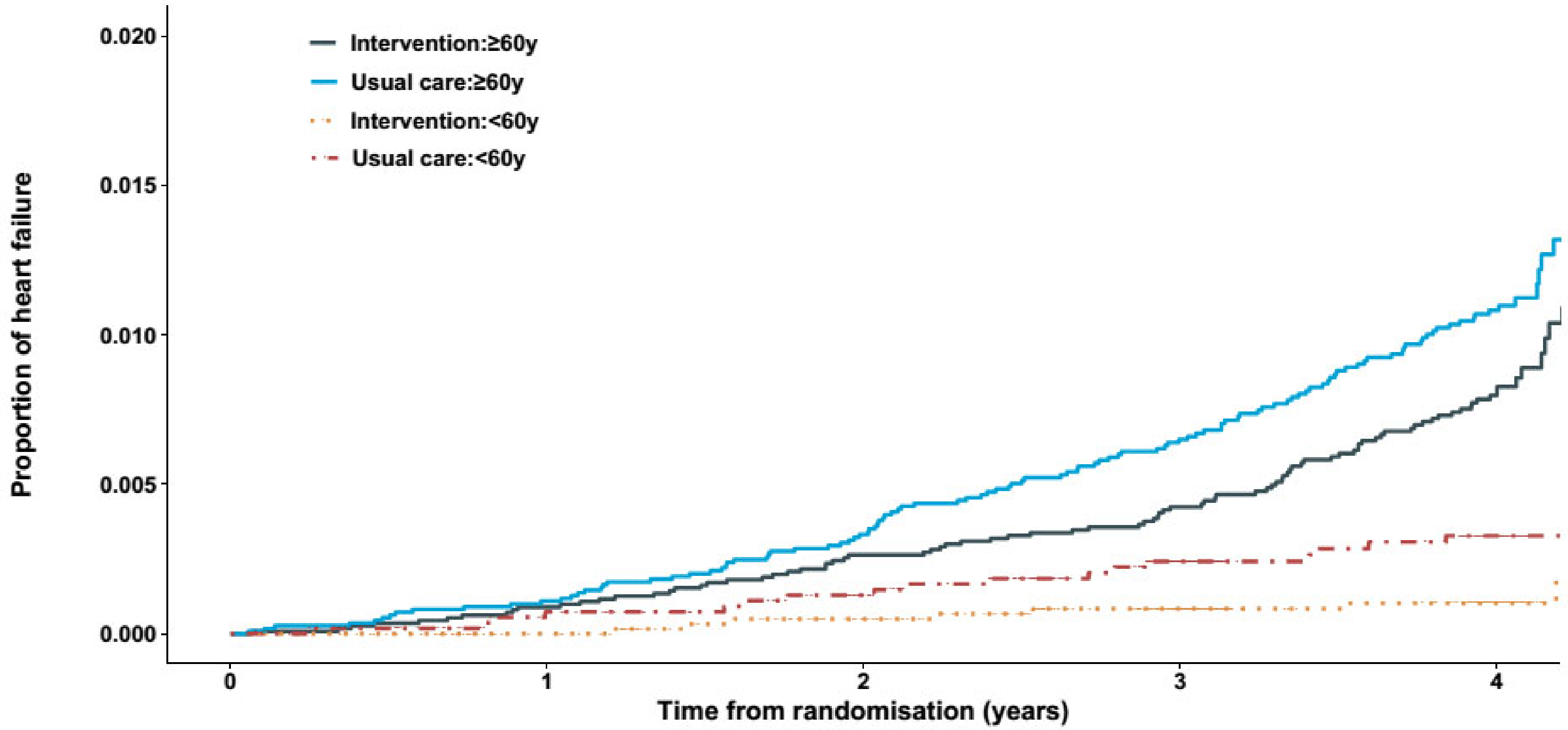

Number at risk

Intervention:≥60y	11258	11052	10789	10078	6752
Intervention:<60y	6092	6044	5979	5652	3965
Usual care:≥60y	11083	10836	10574	9751	6671
Usual care:<60y	5469	5420	5364	5042	3529

eFigure 7: Kaplan-Meier curve for hospitalized heart failure among patients ≥ 60 years and < 60 years.

© 2024 Guo X et al. *JAMA Cardiology*.

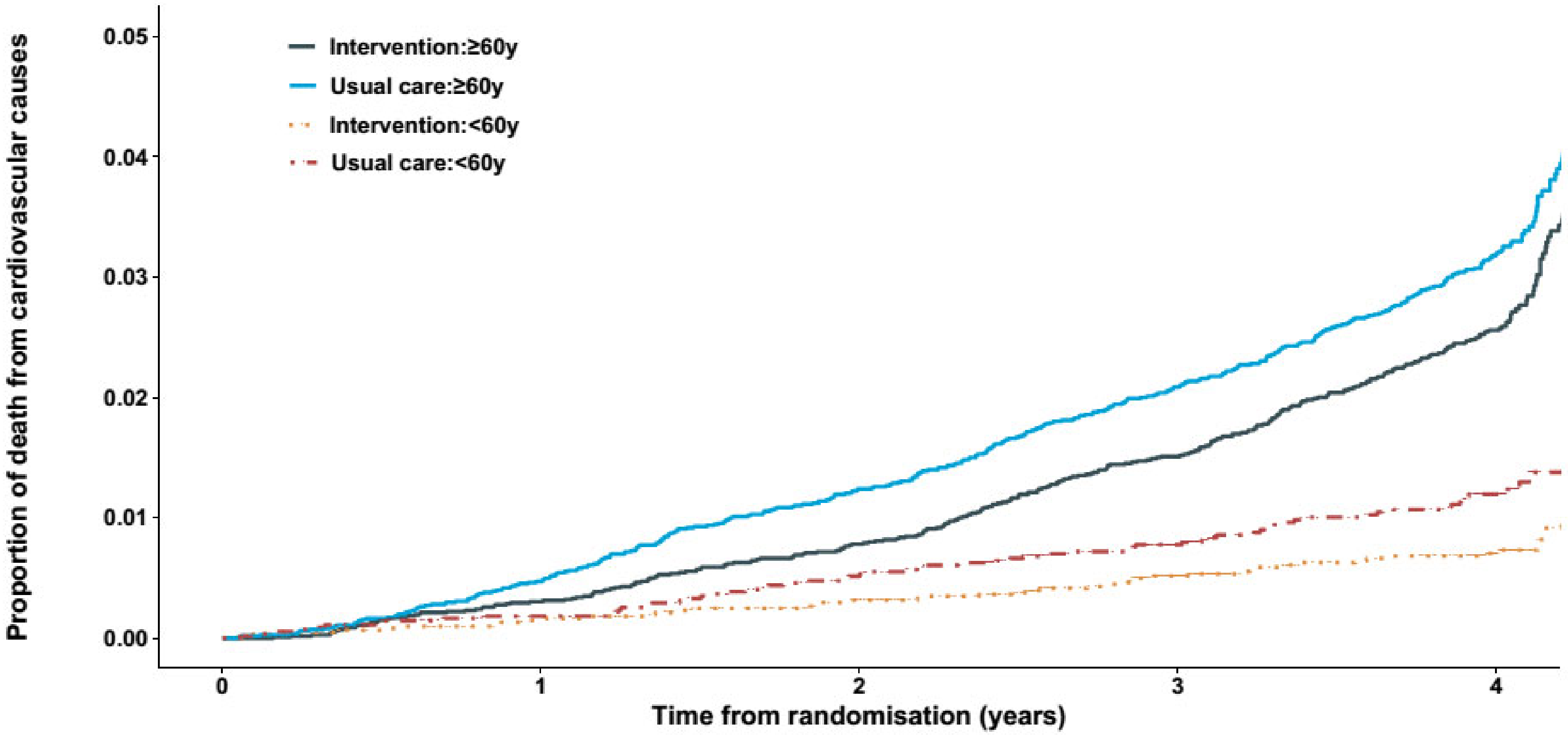

Number at risk

	0	1	2	3	4
Intervention:≥60y	11258	11125	10887	10277	6902
Intervention:<60y	6092	6055	5989	5666	3976
Usual care:≥60y	11083	10927	10675	9948	6865
Usual care:<60y	5469	5436	5376	5075	3556

eFigure 8: Kaplan-Meier curve for death from cardiovascular causes among patients ≥ 60 years and < 60 years.

© 2024 Guo X et al. *JAMA Cardiology*.

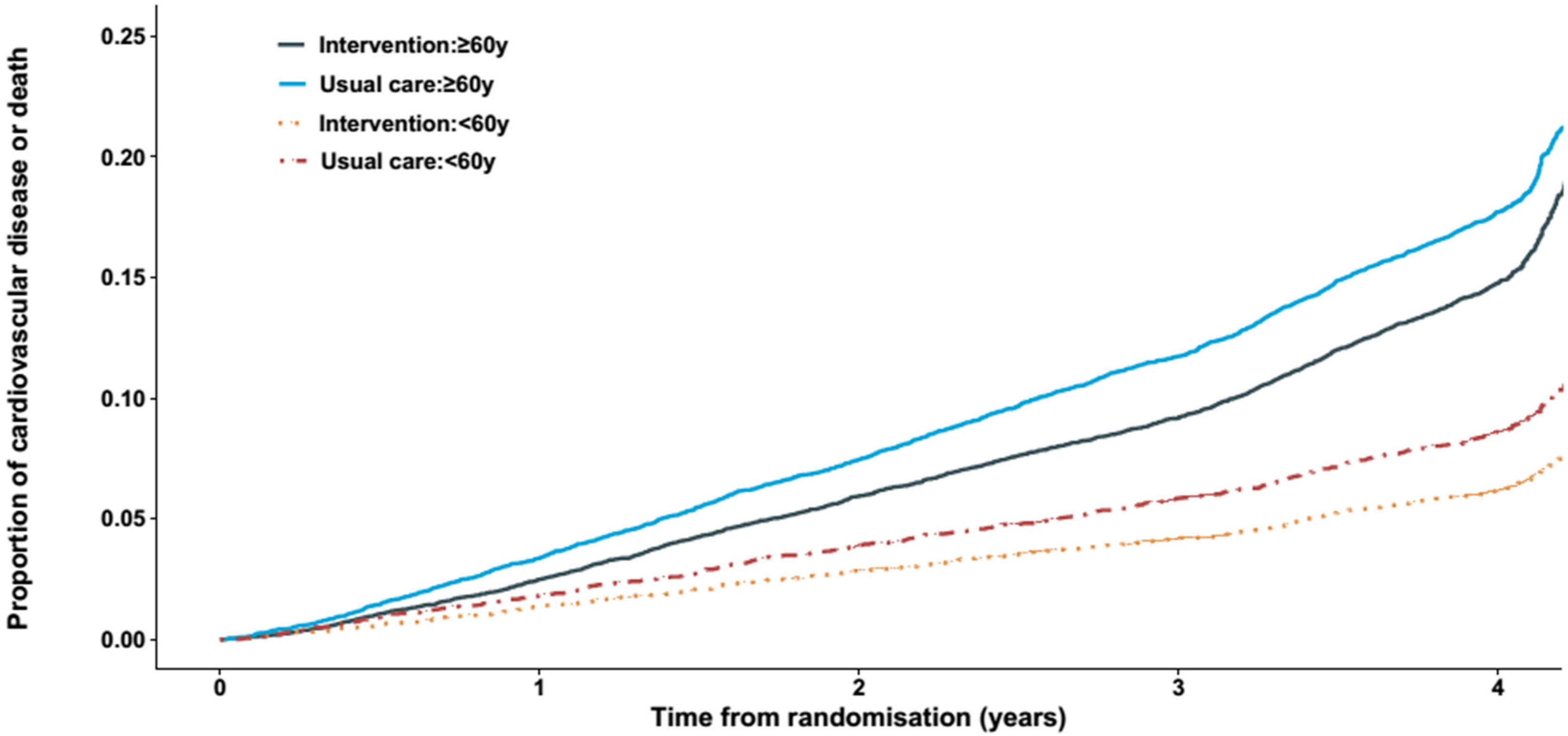

Number at risk

Intervention:≥60y	11258	10964	10556	9857	6507
Intervention:<60y	6092	5997	5882	5529	3849
Usual care:≥60y	11083	10693	10219	9358	6271
Usual care:<60y	5469	5359	5230	4881	3369

eFigure 9: Kaplan-Meier curve for cardiovascular disease or death among patients ≥ 60 years and < 60 years.

© 2024 Guo X et al. *JAMA Cardiology*.

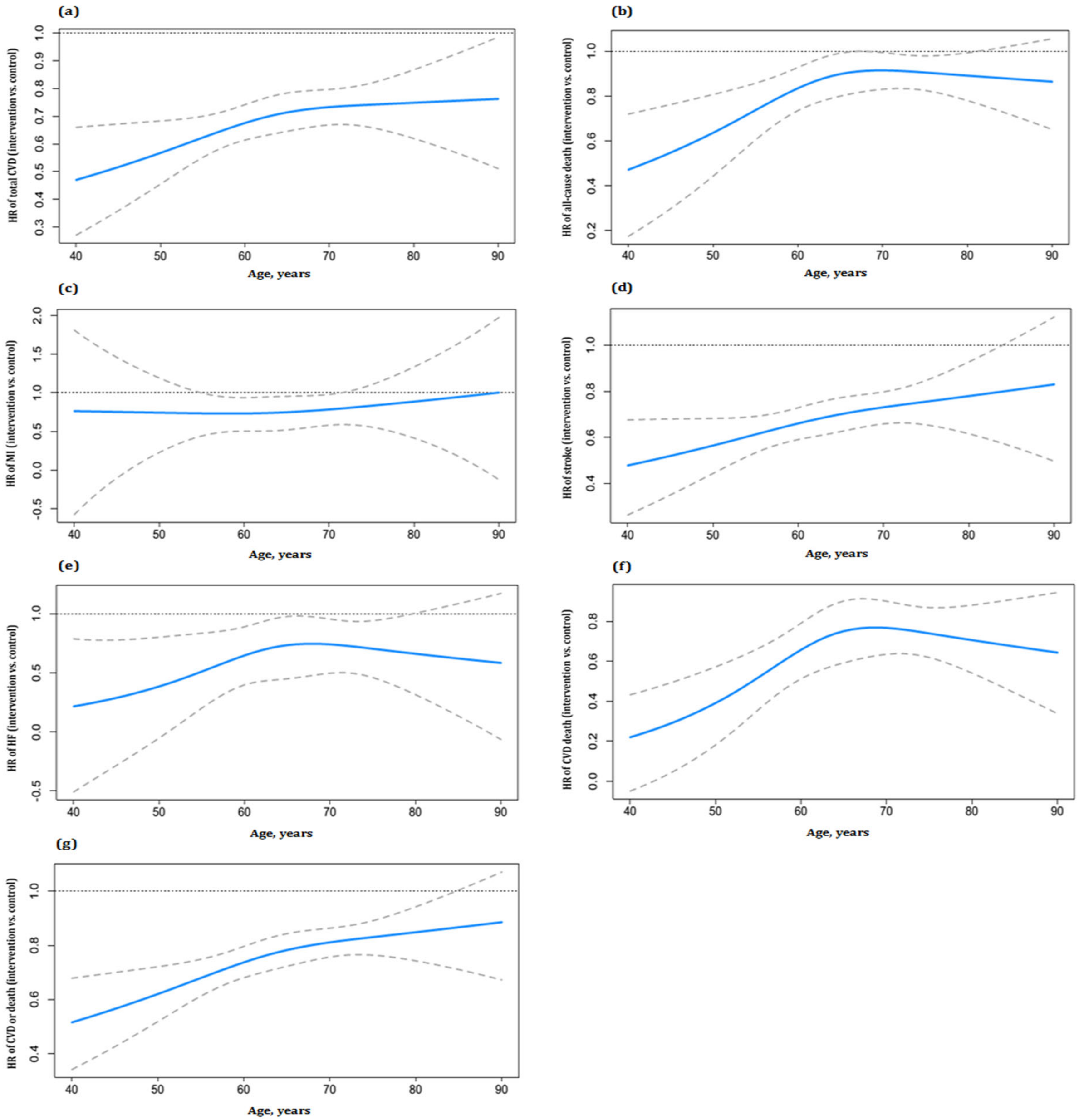

eFigure 10: Risk reductions associated with intervention for each outcome as a function of age. (a) total CVD; (b) all-cause death; (c) MI; (d) stroke; (e) HF; (f) CVD death; (g) total CVD or death. HR=hazard ratio. CVD=cardiovascular disease. MI= myocardial infarction. HF=heart failure.

© 2024 Guo X et al. *JAMA Cardiology*.

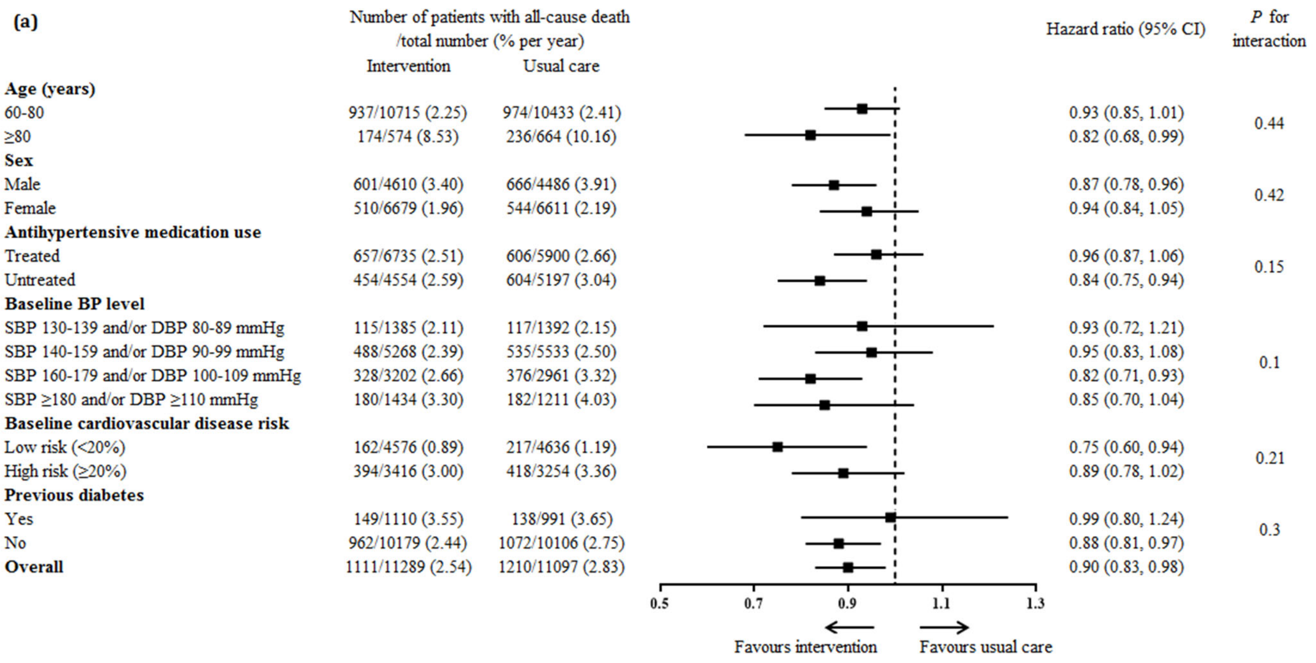

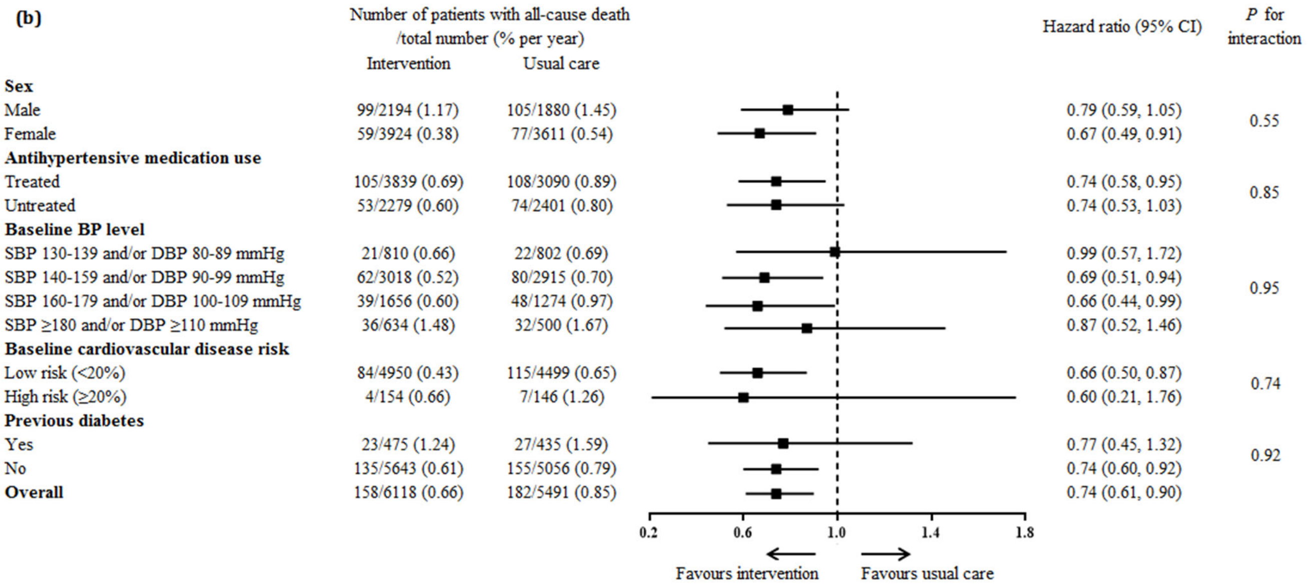

eFigure 11: Forest plot of death from all causes according to subgroups among patients ≥ 60 years (a) and < 60 years (b).

© 2024 Guo X et al. *JAMA Cardiology*.

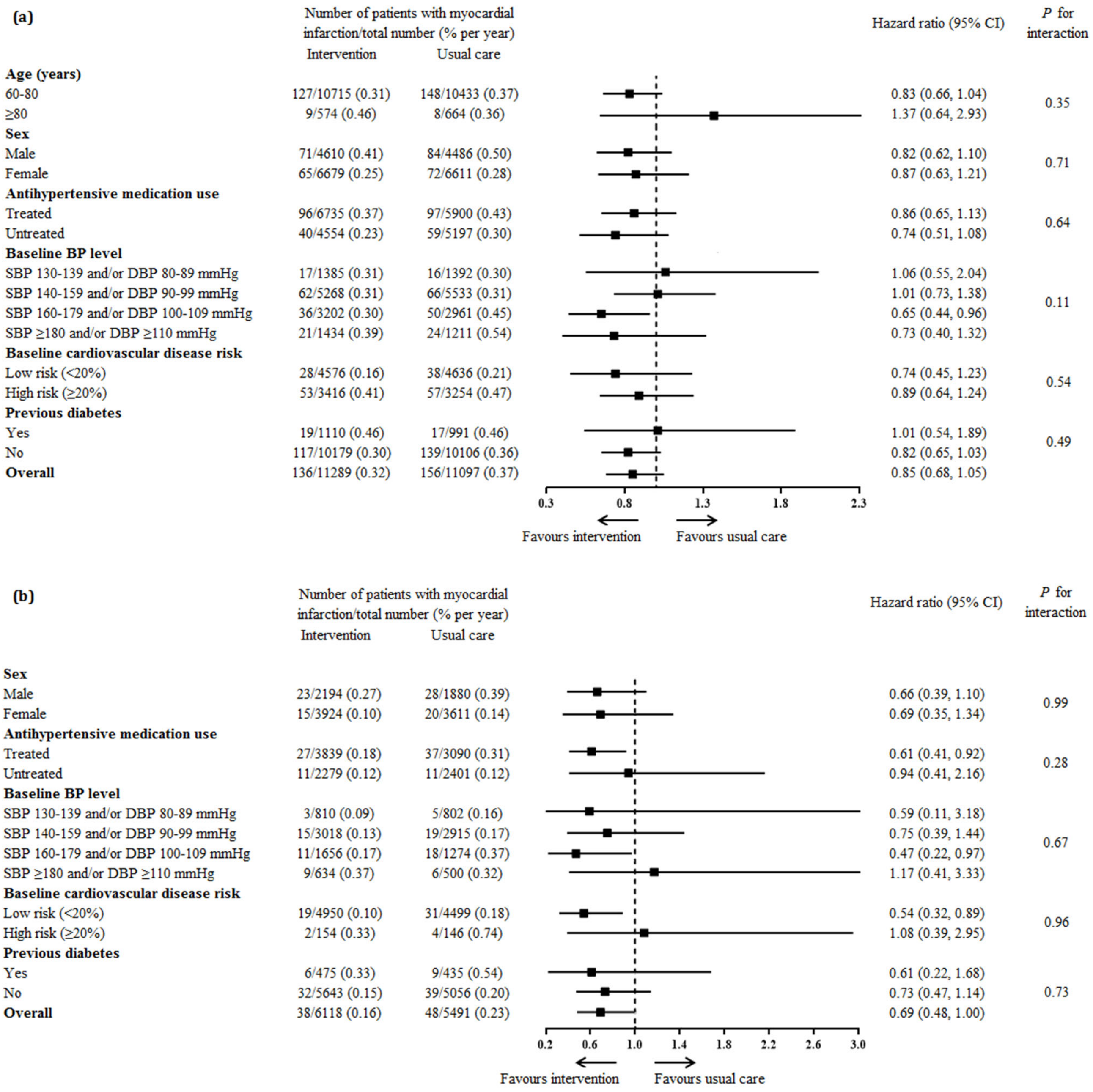

eFigure 12: Forest plot of myocardial infarction according to subgroups among patients ≥ 60 years (a) and < 60 years (b).

© 2024 Guo X et al. *JAMA Cardiology*.

(a)

	Number of patients with stroke/total number (% per year) Intervention	Usual care	Hazard ratio (95% CI)	*P* for interaction
**Age (years)**				
60-80	825/10715 (2.04)	1057/10433 (2.73)	0.73 (0.67, 0.80)	0.18
≥80	57/574 (2.96)	64/664 (2.92)	0.95 (0.67, 1.34)	
**Sex**				
Male	452/4610 (2.66)	588/4486 (3.64)	0.72 (0.64, 0.81)	0.55
Female	430/6679 (1.70)	533/6611 (2.15)	0.77 (0.68, 0.86)	
**Antihypertensive medication use**				
Treated	554/6735 (2.19)	653/5900 (3.00)	0.73 (0.65, 0.81)	0.51
Untreated	328/4554 (1.93)	468/5197 (2.44)	0.77 (0.67, 0.88)	
**Baseline BP level**				
SBP 130-139 and/or DBP 80-89 mmHg	83/1385 (1.56)	113/1392 (2.15)	0.72 (0.54, 0.96)	0.48
SBP 140-159 and/or DBP 90-99 mmHg	365/5268 (1.84)	493/5533 (2.39)	0.75 (0.66, 0.86)	
SBP 160-179 and/or DBP 100-109 mmHg	264/3202 (2.21)	359/2961 (3.33)	0.64 (0.55, 0.74)	
SBP ≥180 and/or DBP ≥110 mmHg	170/1434 (3.26)	156/1211 (3.66)	0.94 (0.76, 1.16)	
**Baseline cardiovascular disease risk**				
Low risk (<20%)	207/4576 (1.16)	284/4636 (1.60)	0.70 (0.58, 0.84)	0.57
High risk (≥20%)	261/3416 (2.05)	357/3254 (3.00)	0.67 (0.57, 0.77)	
**Previous diabetes**				
Yes	132/1110 (3.28)	148/991 (4.17)	0.76 (0.61, 0.94)	0.86
No	750/10179 (1.96)	973/10106 (2.60)	0.74 (0.67, 0.81)	
**Overall**	882/11289 (2.08)	1121/11097 (2.74)	0.74 (0.68, 0.81)	

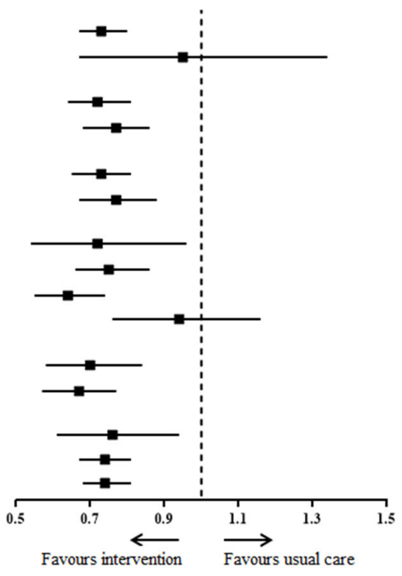

(b)

	Number of patients with stroke/total number (% per year) Intervention	Usual care	Hazard ratio (95% CI)	*P* for interaction
**Sex**				
Male	133/2194 (1.60)	171/1880 (2.44)	0.65 (0.52, 0.80)	0.9
Female	136/3924 (3.47)	192/3611 (5.32)	0.64 (0.52, 0.80)	
**Antihypertensive medication use**				
Treated	193/3839 (1.30)	250/3090 (2.13)	0.60 (0.51, 0.72)	0.46
Untreated	76/2279 (3.34)	113/2401 (4.71)	0.68 (0.50, 0.92)	
**Baseline BP level**				
SBP 130-139 and/or DBP 80-89 mmHg	17/810 (0.53)	45/802 (1.46)	0.33 (0.20, 0.57)	0.56
SBP 140-159 and/or DBP 90-99 mmHg	113/3018 (0.97)	137/2915 (1.22)	0.75 (0.59, 0.96)	
SBP 160-179 and/or DBP 100-109 mmHg	80/1656 (1.26)	114/1274 (2.37)	0.52 (0.40, 0.68)	
SBP ≥180 and/or DBP ≥110 mmHg	59/634 (2.51)	67/500 (3.67)	0.75 (0.51, 1.10)	
**Baseline cardiovascular disease risk**				
Low risk (<20%)	132/4950 (0.69)	207/4499 (1.20)	0.57 (0.46, 0.70)	0.46
High risk (≥20%)	11/154 (1.84)	13/146 (2.39)	0.68 (0.34, 1.39)	
**Previous diabetes**				
Yes	36/475 (2.00)	43/435 (2.61)	0.70 (0.46, 1.06)	0.53
No	233/5643 (1.07)	320/5056 (1.66)	0.64 (0.54, 0.76)	
**Overall**	269/6118 (1.14)	363/5491 (1.74)	0.64 (0.55, 0.76)	

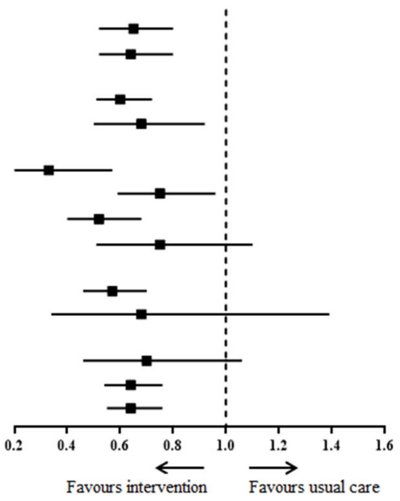

eFigure 13: Forest plot of stroke according to subgroups among patients ≥ 60 years (a) and < 60 years (b).

© 2024 Guo X et al. *JAMA Cardiology*.

(a)

	Number of patients with heart failure/total number (% per year) Intervention	Usual care	Hazard ratio (95% CI)	*P* for interaction
**Age (years)**				
60-80	91/10715 (0.22)	116/10433 (0.29)	0.75 (0.58, 0.96)	0.95
≥80	7/574 (0.36)	11/664 (0.50)	0.72 (0.29, 1.84)	
**Sex**				
Male	55/4610 (0.32)	63/4486 (0.38)	0.79 (0.57, 1.10)	0.39
Female	43/6679 (0.17)	64/6611 (0.25)	0.66 (0.45, 0.95)	
**Antihypertensive medication use**				
Treated	60/6735 (0.23)	83/5900 (0.37)	0.63 (0.46, 0.88)	0.095
Untreated	38/4554 (0.22)	44/5197 (0.22)	0.99 (0.66, 1.50)	
**Baseline BP level**				
SBP 130-139 and/or DBP 80-89 mmHg	10/1385 (0.18)	17/1392 (0.32)	0.52(0.26, 1.08)	0.76
SBP 140-159 and/or DBP 90-99 mmHg	48/5268 (0.24)	59/5533 (0.28)	0.84 (0.60, 1.18)	
SBP 160-179 and/or DBP 100-109 mmHg	27/3202 (0.22)	33/2961 (0.30)	0.75 (0.45, 1.26)	
SBP ≥180 and/or DBP ≥110 mmHg	13/1434 (0.24)	18/1211 (0.41)	0.59 (0.29, 1.21)	
**Baseline cardiovascular disease risk**				
Low risk (<20%)	20/4576 (0.11)	22/4636 (0.12)	0.91 (0.49, 1.69)	0.88
High risk (≥20%)	35/3416 (0.27)	38/3254 (0.31)	0.87 (0.55, 1.37)	
**Previous diabetes**				
Yes	16/1110 (0.39)	19/991 (0.51)	0.70 (0.39, 1.25)	0.98
No	82/10179 (0.21)	108/10106 (0.28)	0.73 (0.55, 0.95)	
**Overall**	98/11289 (0.23)	127/11097 (0.30)	0.73 (0.57, 0.93)	

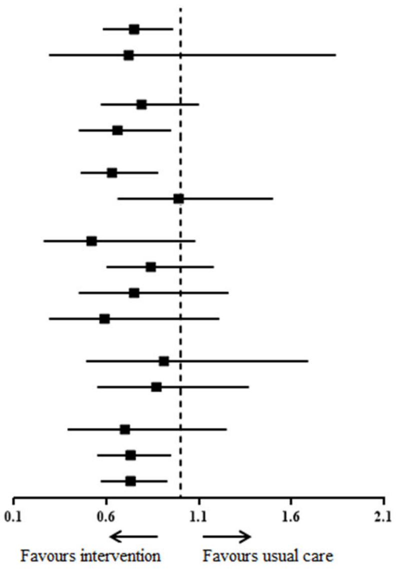

(b)

	Number of patients with heart failure/tota number (% per year) Intervention	Usual care	Hazard ratio (95% CI)	*P* for interaction
**Sex**				
Male	5/2194 (0.06)	7/1880 (0.10)	0.58 (0.19, 1.84)	0.35
Female	4/3924 (0.03)	13/3611 (0.09)	0.28 (0.09, 0.85)	
**Antihypertensive medication use**				
Treated	4/3839 (0.03)	14/3090 (0.12)	0.23 (0.08, 0.68)	0.16
Untreated	5/2279 (0.06)	6/2401 (0.06)	0.87 (0.25, 3.10)	
**Baseline BP level**				
SBP 130-139 and/or DBP 80-89 mmHg	1/810 (0.03)	1/802 (0.03)	0.89 (0.06, 12.65)	0.94
SBP 140-159 and/or DBP 90-99 mmHg	3/3018 (0.03)	8/2915 (0.07)	0.34 (0.09, 1.26)	
SBP 160-179 and/or DBP 100-109 mmHg	2/1656 (0.03)	6/1274 (0.12)	0.26 (0.05, 1.25)	
SBP ≥180 and/or DBP ≥110 mmHg	3/634 (0.12)	5/500 (0.26)	0.48 (0.12, 1.95)	
**Baseline cardiovascular disease risk**				
Low risk (<20%)	3/4950 (0.02)	11/4499 (0.06)	0.24 (0.06, 0.90)	NA
High risk (≥20%)	0/154 (0)	2/146 (0.36)	NA	
**Previous diabetes**				
Yes	4/475 (0.22)	3/435 (0.18)	1.15 (0.27, 4.80)	0.1
No	5/5643 (0.02)	17/5056 (0.09)	0.26 (0.09, 0.70)	
**Overall**	9/6118 (0.04)	20/5491 (0.09)	0.39 (0.18, 0.87)	

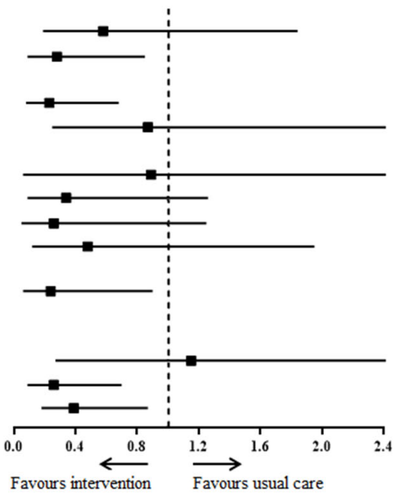

eFigure 14: Forest plot of hospitalized heart failure according to subgroups among patients ≥ 60 years (a) and < 60 years (b).

© 2024 Guo X et al. *JAMA Cardiology*.

(a)

	Number of patients with cardiovascular death/total number (% per year) Intervention	Usual care	Hazard ratio (95% CI)	*P* for interaction
**Age (years)**				
60-80	267/10715 (0.64)	343/10433 (0.85)	0.74 (0.64, 0.85)	0.37
≥80	48/574 (2.35)	63/664 (2.70)	0.89 (0.62, 1.27)	
**Sex**				
Male	172/4610 (0.97)	223/4486 (1.31)	0.73 (0.62, 0.86)	0.84
Female	143/6679 (0.55)	183/6611 (0.71)	0.77 (0.62, 0.95)	
**Antihypertensive medication use**				
Treated	202/6735 (0.77)	226/5900 (0.99)	0.78 (0.66, 0.92)	0.47
Untreated	113/4554 (0.65)	180/5197 (0.90)	0.70 (0.57, 0.86)	
**Baseline BP level**				
SBP 130-139 and/or DBP 80-89 mmHg	34/1385 (0.62)	34/1392 (0.62)	0.90 (0.60, 1.35)	0.25
SBP 140-159 and/or DBP 90-99 mmHg	130/5268 (0.64)	167/5533 (0.78)	0.80 (0.65, 0.99)	
SBP 160-179 and/or DBP 100-109 mmHg	90/3202 (0.73)	142/2961 (1.25)	0.57 (0.44, 0.74)	
SBP ≥180 and/or DBP ≥110 mmHg	61/1434 (1.12)	63/1211 (1.39)	0.88 (0.62, 1.25)	
**Baseline cardiovascular disease risk**				
Low risk (<20%)	38/4576 (0.21)	61/4636 (0.33)	0.63 (0.41, 0.96)	0.75
High risk (≥20%)	94/3416 (0.72)	130/3254 (1.04)	0.68 (0.52, 0.88)	
**Previous diabetes**				
Yes	50/1110 (1.19)	59/991 (1.56)	0.73 (0.52, 1.03)	0.98
No	265/10179 (0.67)	347/10106 (0.89)	0.74 (0.64, 0.86)	
**Overall**	315/11289 (0.72)	406/11097 (0.95)	0.75 (0.65, 0.85)	

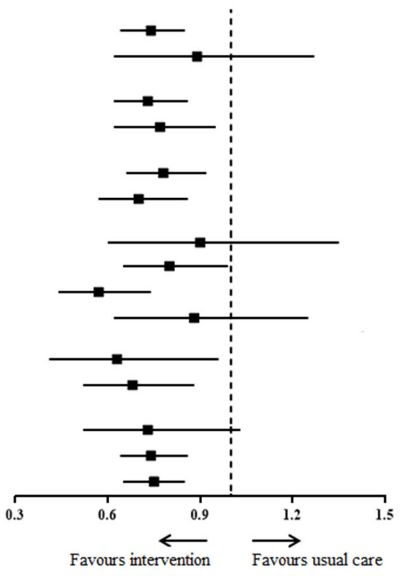

(b)

	Number of patients with cardiovascular death/total number (% per year) Intervention	Usual care	Hazard ratio (95% CI)	*P* for interaction
**Sex**				
Male	33/2194 (0.39)	50/1880 (0.69)	0.57 (0.37, 0.88)	0.84
Female	15/3924 (0.10)	27/3611 (0.19)	0.49 (0.27, 0.89)	
**Antihypertensive medication use**				
Treated	29/3839 (0.19)	50/3090 (0.41)	0.46 (0.29, 0.71)	0.24
Untreated	19/2279 (0.21)	27/2401 (0.29)	0.70 (0.39, 1.26)	
**Baseline BP level**				
SBP 130-139 and/or DBP 80-89 mmHg	4/810 (0.12)	11/802 (0.35)	0.35 (0.10, 1.19)	0.23
SBP 140-159 and/or DBP 90-99 mmHg	14/3018 (0.12)	30/2915 (0.26)	0.42 (0.23, 0.76)	
SBP 160-179 and/or DBP 100-109 mmHg	16/1656 (0.25)	21/1274 (0.42)	0.60 (0.31, 1.16)	
SBP ≥180 and/or DBP ≥110 mmHg	14/634 (0.57)	15/500 (0.78)	0.75 (0.33, 1.71)	
**Baseline cardiovascular disease risk**				
Low risk (<20%)	15/4950 (0.08)	38/4499 (0.22)	0.36 (0.20, 0.64)	0.34
High risk (≥20%)	2/154 (0.33)	2/146 (0.36)	0.75 (0.12, 4.49)	
**Previous diabetes**				
Yes	7/475 (0.38)	13/435 (0.76)	0.59 (0.22, 1.57)	0.75
No	41/5643 (0.73)	64/5056 (1.27)	0.55 (0.37, 0.82)	
**Overall**	48/6118 (0.78)	77/5491 (1.40)	0.54 (0.37, 0.77)	

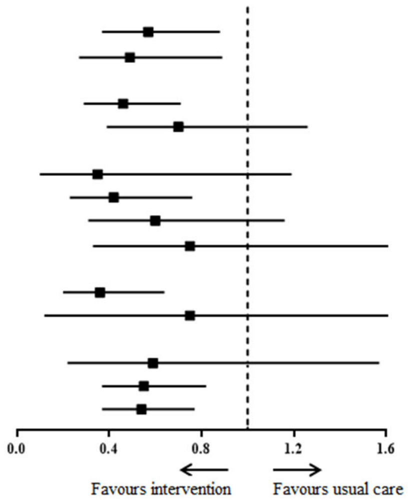

eFigure 15: Forest plot of death from cardiovascular causes according to subgroups among patients ≥ 60 years (a) and < 60 years (b).

© 2024 Guo X et al. *JAMA Cardiology*.

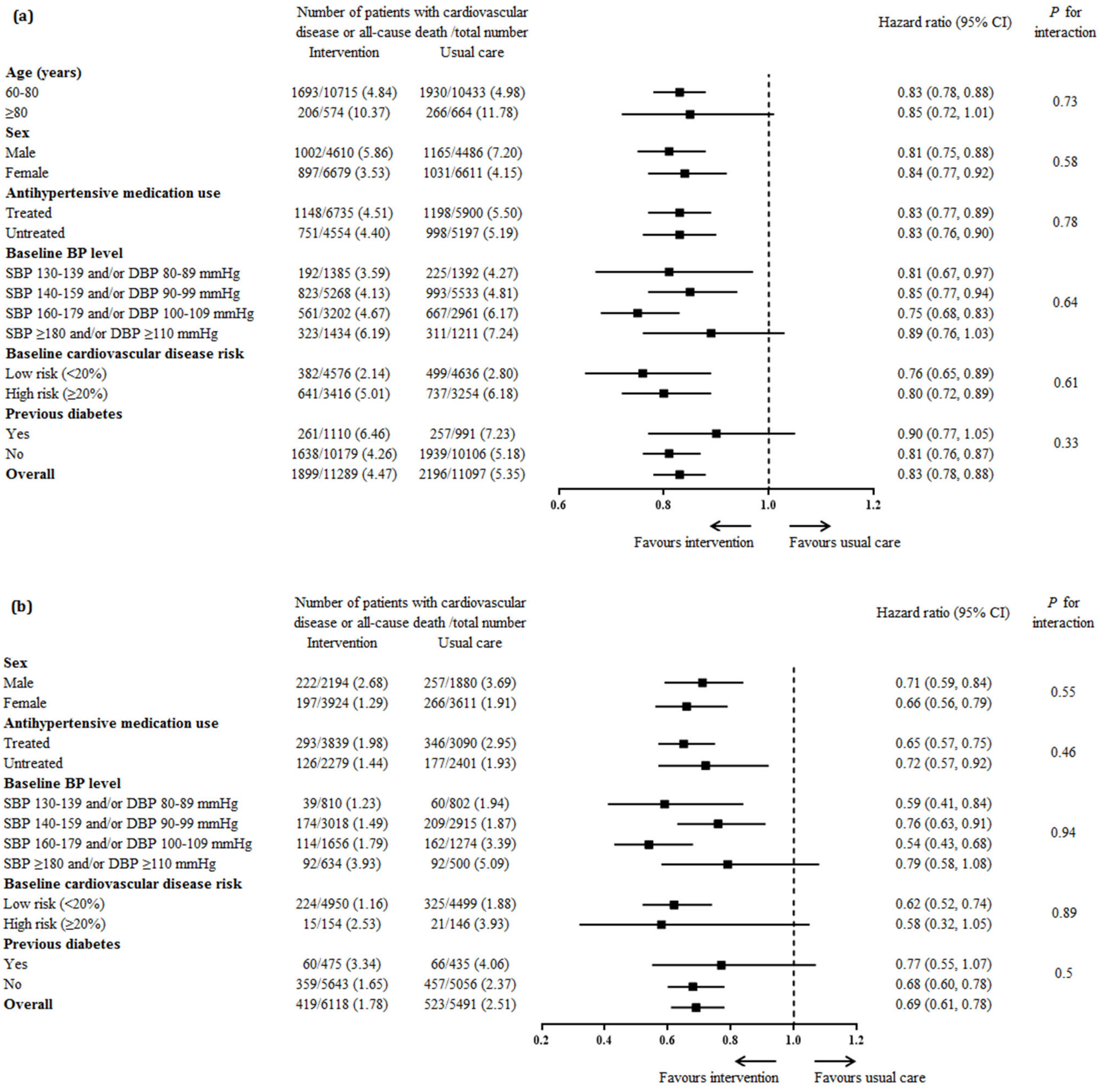

eFigure 16: Forest plot of cardiovascular disease or death according to subgroups among patients ≥ 60 years (a) and < 60 years (b).

© 2024 Guo X et al. *JAMA Cardiology*.

eTable 1: Implementation strategies

**Intervention on healthcare systems**<br>• We established an organizational structure at city/county, town and village levels to collaboratively manage hypertension at the beginning of the study. The non-physician community healthcare practitioners in villages were responsible for routine management of hypertensive patient; primary care physicians at township hospitals guided and audited blood pressure management and provided monthly feedback to the community healthcare practitioners; hypertension specialists at city/county hospitals provided training to community healthcare practitioners and helped timely in managing patients with complications.<br>• Using hypertension control rate as the core index, non-physician community health-care practitioners received a portion of their salaries from research grant funds for study-related activities and performance-based incentive supplement to enhance hypertension management performance throughout the whole intervention.<br>• Covered by research grants, hypertensive patients received discounted or free antihypertensive medications monthly from non-physician community health-care practitioners to achieve better blood pressure control.
**Intervention on non-physician community healthcare** practitioner**s**<br>• Non-physician community healthcare practitioners received training on how to measure blood pressure according to a standard protocol to ensure the validity of the blood pressure data at early stage of the study.<br>• Non-physician community healthcare practitioners received a series of trainings on how to use a simple stepwise protocol for hypertension treatment to enhance hypertension management performance periodically throughout the whole intervention.<br>• Non-physician community healthcare practitioners received a series of trainings on how to conduct health coaching on lifestyle change (e·g·, reducing sodium and alcohol intake) and improve medication adherence periodically throughout the study.
**Intervention on patients**<br>• Hypertensive patients received free home blood pressure monitors and instructions on measuring their blood pressure 2-3 days a week to enhance medication adherence at the beginning of the study.<br>• Hypertensive patients received health coaching at individual visits or group sessions monthly in the first 6 months and quarterly thereafter throughout the study.<br>• Social support groups among patients and family members were established in each intervention village, and they met in-person or through social media (i.e., WeChat) and telephone throughout the study.

© 2024 Guo X et al. *JAMA Cardiology*.

eTable 2: Characteristics of clusters in the China Rural Hypertension Control Project.

	Intervention	Usual care
No. of villages	163	163
No. of townships	46	46
No. of counties	13	13
Mean no. of residents per village at baseline (SD)*	1540 (635)	1504 (631)
Mean no. of residents aged ≥40 years per village at baseline (SD)*	827 (397)	803 (357)
Mean no. of registered hypertension patients per village at baseline (SD)	295 (119)	272 (105)
Mean no. of study participants per village at baseline (SD)	107 (21)<br>(range: 32-141)	102 (20)<br>(range: 40-145)
No. of villages with two or more non-physician community healthcare practitioners, n (%)	27 (16·6)	21 (12·9)
Mean distance to township hospitals (SD), km	6·0 (4·0)	6·3 (4·6)

SD = standard deviation.
* Number of residents who lived in village during the baseline examination. The migrant workers who did not live in the villages during baseline examination were excluded.

© 2024 Guo X et al. *JAMA Cardiology*.

eTable 3: Intraclass correlation coefficients (95% confidence intervals) of cardiovascular and mortality outcomes during 48-month follow-up.

	Overall	Intervention	Usual care
**Patients aged ≥ 60 years**			
Cardiovascular disease (myocardial infarction, stroke, heart failure, or cardiovascular disease death)	0.0072 (0.0038, 0.0105)	0.0023 (0.0000, 0.0059)	0.0076 (0.0028, 0.0124)
Myocardial infarction	0.0004 (0.0000, 0.0027)	NA	0.0017 (0.0000, 0.0053)
Stroke	0.0083 (0.0048, 0.0118)	0.0055 (0.0012,0.0099)	0.0078 (0.0029, 0.0126)
Heart failure	0.0014 (0.0000, 0.0038)	0.0023 (0.0000, 0.0059)	0.0004 (0.0000, 0.0037)
Death from cardiovascular causes	0.0010 (0.0000, 0.0034)	NA	0.0012 (0.0000, 0.0047)
Death from all causes	0.0053 (0.0022, 0.0084)	0.0047 (0.0005, 0.0089)	0.0054 (0.0010, 0.0098)
Cardiovascular disease or death	0.0051 (0.0021, 0.0081)	0.0005 (0.0000, 0.0038)	0.0065 (0.0019, 0.0111)
**Patients aged < 60 years**			
Cardiovascular disease (myocardial infarction, stroke, heart failure, or cardiovascular disease death)	0.0103 (0.0044, 0.0162)	0.0033 (0.0000, 0.0099)	0.0103 (0.0016, 0.0191)
Myocardial infarction	NA	NA	NA
Stroke	0.0093 (0.0035, 0.0151)	0.0045 (0.0000, 0.0113)	0.0085 (0.0001, 0.0168)
Heart failure	0.0008 (0.0000, 0.0053)	NA	0.0009 (0.0000, 0.0077)
Death from cardiovascular causes	0.0050 (0.0000, 0.0101)	0.0050 (0.0000, 0.0119)	0.0030 (0.0000, 0.0102)
Death from all causes	0.0039 (0.0000, 0.0088)	0.0007 (0.0000, 0.0068)	0.0056 (0.0000, 0.0133)
Cardiovascular disease or death	0.0088 (0.0031, 0.0144)	0.0039 (0.0000, 0.0106)	0.0082 (0.0000, 0.0165)

NA: Smith's Large Sample Confidence Interval for ICC is Not Estimable.

© 2024 Guo X et al. *JAMA Cardiology*.

eTable 4: Effectiveness of a non-physician community health-care practitioner-led intervention on cardiovascular and mortality outcomes among patients aged ≥ 80 years.

	Intervention		Usual care		Hazard ratio (95% CI)	p value*	Multiple-adjusted hazard ratio (95% CI)	p value†
	Number of events	Rate per year	Number of events	Rate per year				
Cardiovascular disease (myocardial infarction, stroke, heart failure, or cardiovascular disease death)	83	4.3%	103	4.7%	0.91 (0.70, 1.19)	0.49	0.91 (0.70, 1.19)	0.5
Myocardial infarction	9	0.5%	8	0.4%	1.37 (0.64, 2.93)	0.42	1.03 (0.44, 2.44)	0.95
Stroke	57	3.0%	64	2.9%	0.95 (0.67, 1.34)	0.75	0.90 (0.64, 1.27)	0.55
Heart failure	7	0.4%	11	0.5%	0.72 (0.29, 1.84)	0.5	0.75 (0.34, 1.63)	0.47
Death from cardiovascular causes	48	2.4%	63	2.7%	0.89 (0.62, 1.27)	0.51	0.93 (0.65, 1.33)	0.68
Death from all causes	174	8.5%	236	10.2%	0.82 (0.68, 0.99)	0.03	0.88 (0.73, 1.06)	0.17
Cardiovascular disease or death	206	10.4%	266	11.8%	0.85 (0.72, 1.01)	0.06	0.88 (0.74, 1.05)	0.15

CIs were not adjusted for multiple comparisons and should not be used in place of hypothesis testing. *In the marginal Cox models, village was used as a random effect and the stratification variables (province, county, and township) as fixed effects. †Additionally adjusted for age, sex, cigarette smoking, use of antihypertensive medication, history of cardiovascular disease, and baseline systolic blood pressure, low-density lipoprotein cholesterol, and fasting plasma glucose.

© 2024 Guo X et al. *JAMA Cardiology*.

eTable 5: Safety and kidney outcomes by randomization groups among patients aged ≥ 80 years.

	Intervention (n=574)	Usual care (n=664)	Risk ratio (95% CI)	p value
Conditions of interest				
Injurious falls*	1 (0.2)	2 (0.3)	0.58 (0.01, 11.11)	0.71
Hypotension†	14 (2.4)	3 (0.5)	5.40 (1.51, 29.30)	<0.01
Symptomatic hypotension‡	3 (0.5)	2 (0.3)	1.74 (0.20, 20.78)	0.58
Syncope§	4 (0.7)	1 (0.2)	4.63 (0.46, 227.88)	0.17
Electrolytes¶				
Serum sodium <130 mmol/L	0 (0)	1 (0.2)	NA	NA
Serum sodium >150 mmol/L	1 (0.2)	8 (1.7)	0.14 (0, 1.02)	0.03
Serum potassium <3·0 mmol/L	0	0	NA	NA
Serum potassium >5·5 mmol/L	8 (1.8)	3 (0.6)	2.93 (0.70, 17.12)	0.11
Renal outcomes¶				
≥50% reduction in estimated GFR in patients with chronic kidney disease at baseline‖	1/33 (3.0)	0/48 (0)	NA	NA
≥30% reduction in estimated GFR to <60 mL/min per 1·73 m² in patients without chronic kidney disease at baseline‖	21/400 (5.3)	18/427 (4.2)	1.25 (0.63, 2.48)	0.5

Data are n (%) or n/N (%). GFR=glomerular filtration rate. *Self-reported injurious fall was defined as a fall that resulted in seeking medical care in a hospital, a primary care clinic, or a village doctor's office. †Systolic blood pressure less than 90 mm Hg at a village doctor visit or a study data collection visit at months 6, 12, 18, 24, 30, 36 and 48. ‡Self-reported symptomatic hypotension was confirmed by systolic blood pressure less than 90 mm Hg at a village doctor visit. §Self-reported temporary loss of consciousness that resulted in seeking medical care in a hospital, a primary care clinic, or a village doctor's office. ¶Results were based on 36 month follow-up. ‖Estimated GFR was calculated using the 2021 Chronic Kidney Disease Epidemiology Collaboration creatinine equations. Chronic kidney disease at baseline was defined as estimated GFR of less than 60 mL/min per 1·73 m².

© 2024 Guo X et al. *JAMA Cardiology*.
