# Supplementary material for: Multifaceted Intensive Blood Pressure Control Model in Older and Younger Individuals With Hypertension: A Randomized Clinical Trial
Source: JAMA Cardiol. 2024 Jun 18;9(9):781–90. doi: 10.1001/jamacardio.2024.1449 (PMC11195599; doi:10.1001/jamacardio.2024.1449)
Supplement: Supplement 4. — Nonauthor Collaborators. CRHCP Study Group. [file jamacardiol-e241449-s004.pdf]

Supplemental Online Content: Nonauthor Collaborators

*Indicates required information. Only first name, last name, and suffix will appear in PubMed.

*Group Name(s): CRHCP Study Group							
***First Name and Middle Initial(s)**	***Last Name**	***Suffix (eg, Jr, III)**	Academic Degrees	Institution	Location (city, state/province, country)	Role or Contribution, eg, chair, principal investigator	Group (if more than 1 Group listed in the byline) and/or Subgroup (eg, Steering Committee)
Jiang	He						
Jianjun	Mu						
Dao Wen	Wang						
Liying	Xing						
Guocheng	Ren						
Chunxia	Zhao						
Ruihai	Yang						
Chung-Shiuan	Chen						
Jun	Wang						
Ning	Ye						
Zihao	Fan						
Nan	Ye						
Linlin	Zhang						
Shu	Zhang						
Qiyu	Li						
Qiying	Qin						
Canru	Liu						
Xiaoyu	Zheng						
Tao	Wang						
Li	Jing						
Boqiang	Zhang						
Qun	Sun						
Yu	Yan						
Yueyuan	Liao						
Qiong	Ma						
Chao	Chu						
Yue	Sun						
Dan	Wang						
Ling	Zhou						

*Indicates required information. Only first name, last name, and suffix will appear in PubMed.

*First Name and Middle Initial(s)	*Last Name	*Suffix (eg, Jr, III)	Academic Degrees	Institution	Location (city, state/province, country)	Role or Contribution, eg, chair, principal investigator	Group (if more than 1 Group listed in the byline) and/or Subgroup (eg, Steering Committee)
Heng	Ye						
Haoran	Wei						
Hao	Liu						
Zhaoqing	Sun						
Liqiang	Zheng						
Yanli	Chen						
Ye	Chang						
Mohan	Jiang						
Hongmei	Yang						
Shasha	Yu						
Wenna	Li						
Ning	Wang						
Chunwei	Wu						
Lufan	Sun						
Zhi	Du						
Yan	Li						
Nan	Gao						
Xinchi	Liu						
Ying	Wang						
Mingang	Huang						
Yufang	Zhou						
Lingrui	Meng						
Jiawen	Zhang						
Zhen	Huang						
Huihui	Chen						
Yuxian	Huang						
Lingmin	Sun						
Xin	Zhong						
Hanmin	Wang						
Xinyan	Hou						
Huan	Han						

Supplemental Online Content: Nonauthor Collaborators

*Indicates required information. Only first name, last name, and suffix will appear in PubMed.

***First Name and Middle Initial(s)**	***Last Name**	***Suffix (eg, Jr, III)**	Academic Degrees	Institution	Location (city, state/province, country)	Role or Contribution, eg, chair, principal investigator	Group (if more than 1 Group listed in the byline) and/or Subgroup (eg, Steering Committee)
Baohui	Jin						
