# Supplementary material for: Multifaceted Intensive Blood Pressure Control Model in Older and Younger Individuals With Hypertension: A Randomized Clinical Trial
Source: JAMA Cardiol. 2024 Jun 18;9(9):781–90. doi: 10.1001/jamacardio.2024.1449 (PMC11195599; doi:10.1001/jamacardio.2024.1449)
Supplement: Supplement 5. — Data Sharing Statement. [file jamacardiol-e241449-s005.pdf]

Data Sharing Statement

Guo. Multifaceted Intensive Blood Pressure Control Model in Older and Younger Individuals With Hypertension. *JAMA Cardiol*. Published June 18, 2024.
doi:10.1001/jamacardio.2024.1449

Data
Data available: Yes
Data types: Deidentified participant data, Data dictionary
How to access data: Data from this study can be requested from Prof Yingxian Sun (yxsun@cmu.edu.cn)
When available: With publication

Supporting Documents
Document types: None

Additional Information
Who can access the data: Specific requests for data will require the submission of a proposal with a valuable research question as assessed by the study steering committee and require a data access agreement to be signed.
Types of analyses: Any analyses with a valuable research question.
Mechanisms of data availability: After approval of a proposal as assessed by the study steering committee and with a signed data access agreement.
